# *MarrowQuant* Across Aging and Aplasia: A Digital Pathology Workflow for Quantification of Bone Marrow Compartments in Histological Sections

**DOI:** 10.3389/fendo.2020.00480

**Published:** 2020-09-24

**Authors:** Josefine Tratwal, David Bekri, Chiheb Boussema, Rita Sarkis, Nicolas Kunz, Tereza Koliqi, Shanti Rojas-Sutterlin, Frédérica Schyrr, Daniel Naveed Tavakol, Vasco Campos, Erica L. Scheller, Rossella Sarro, Carmen Bárcena, Bettina Bisig, Valentina Nardi, Laurence de Leval, Olivier Burri, Olaia Naveiras

**Affiliations:** ^1^Laboratory of Regenerative Hematopoiesis, Institute of Bioengineering and Institute for Experimental Cancer Research, Ecole Polytechnique Fédérale de Lausanne (EPFL), Lausanne, Switzerland; ^2^Animal Imaging and Technology Core, Center for Biomedical Imaging, Ecole Polytechnique Fédérale de Lausanne (EPFL), Lausanne, Switzerland; ^3^Division of Bone and Mineral Diseases, Department of Internal Medicine, Washington University, Saint Louis, MO, United States; ^4^Institute of Pathology, Lausanne University Hospital (CHUV), Lausanne University (UNIL), Lausanne, Switzerland; ^5^Department of Pathology, University Hospital 12 de Octubre, Madrid, Spain; ^6^Department of Pathology, Massachusetts General Hospital, Harvard Medical School, Boston, MA, United States; ^7^Bioimaging and Optics Core Facility, Ecole Polytechnique Fédérale de Lausanne (EPFL), Lausanne, Switzerland; ^8^Department of Oncology, Hematology Service, Lausanne University Hospital (CHUV), Lausanne, Switzerland

**Keywords:** bone marrow, hematoxylin and eosin, histology, adipocyte, pathology, skeleton, cellularity, hematopoietic

## Abstract

The bone marrow (BM) exists heterogeneously as hematopoietic/red or adipocytic/yellow marrow depending on skeletal location, age, and physiological condition. Mouse models and patients undergoing radio/chemotherapy or suffering acute BM failure endure rapid adipocytic conversion of the marrow microenvironment, the so-called “red-to-yellow” transition. Following hematopoietic recovery, such as upon BM transplantation, a “yellow-to-red” transition occurs and functional hematopoiesis is restored. Gold Standards to estimate BM cellular composition are pathologists' assessment of hematopoietic cellularity in hematoxylin and eosin (H&E) stained histological sections as well as volumetric measurements of marrow adiposity with contrast-enhanced micro-computerized tomography (CE-μCT) upon osmium-tetroxide lipid staining. Due to user-dependent variables, reproducibility in longitudinal studies is a challenge for both methods. Here we report the development of a semi-automated image analysis plug-in, *MarrowQuant*, which employs the open-source software QuPath, to systematically quantify multiple bone components in H&E sections in an unbiased manner. *MarrowQuant* discerns and quantifies the areas occupied by bone, adipocyte ghosts, hematopoietic cells, and the interstitial/microvascular compartment. A separate feature, *AdipoQuant*, fragments adipocyte ghosts in H&E-stained sections of extramedullary adipose tissue to render adipocyte area and size distribution. Quantification of BM hematopoietic cellularity with *MarrowQuant* lies within the range of scoring by four independent pathologists, while quantification of the total adipocyte area in whole bone sections compares with volumetric measurements. Employing our tool, we were able to develop a standardized map of BM hematopoietic cellularity and adiposity in mid-sections of murine C57BL/6 bones in homeostatic conditions, including quantification of the highly predictable red-to-yellow transitions in the proximal section of the caudal tail and in the proximal-to-distal tibia. Additionally, we present a comparative skeletal map induced by lethal irradiation, with longitudinal quantification of the “red-to-yellow-to-red” transition over 2 months in C57BL/6 femurs and tibiae. We find that, following BM transplantation, BM adiposity inversely correlates with kinetics of hematopoietic recovery and that a proximal to distal gradient is conserved. Analysis of *in vivo* recovery through magnetic resonance imaging (MRI) reveals comparable kinetics. On human trephine biopsies *MarrowQuant* successfully recognizes the BM compartments, opening avenues for its application in experimental, or clinical contexts that require standardized human BM evaluation.

## Introduction

Bone marrow adipocytes (BMAds) were long considered as passive fillers of the marrow cavity. In recent years, they have become accepted as occupying an important role within the bone marrow (BM) microenvironment in health and disease, while also contributing to whole-body energy homeostasis (1, 2).

At birth, the murine and human skeleton is entirely hematopoietic. BMAds appear shortly thereafter and increase throughout juvenile development in a centripetal fashion as the skeleton matures (3). This reciprocal relationship between adipocytic and hematopoietic content has been known since the enunciation of the “Neumann” law in 1902, describing the age-driven adipocytic conversion of the marrow in distal bones. Indeed, due to the macroscopic coloration of the predominant cell types, the marrow has been broadly categorized as hematopoietic/red or adipocytic/yellow marrow (4). In humans, adipocytes become the most abundant cellular component in the adult BM. In mice, age-dependent adipocytic conversion of the marrow is highly strain-dependent (5). Notably, the C57BL/6 murine strain, which constitutes the most widely used model for experimental hematopoiesis, presents the lowest degree of BM adipocyte content upon homeostatic skeletal maturation. However, upon hematopoietic ablation (e.g., after chemo- or radiotherapy) a massive adipocytic conversion has been consistently described in humans and across different mouse strains. The BM is thus heterogeneous depending on specific skeletal location, age, and physiological condition [reviewed in (6)].

At the single-cell level, however, BMAd heterogeneity was first described by the terms “labile” and “stable” (7). These terms were coined upon the discovery that performic acid Schiff (PFAS) positive and negative stains respectively differentiate “labile” BMAds interspersed within the hematopoietic red BM, which respond to hematopoietic demand (PFAS positive) from “stable” BMAds comprising the non-hematopoietic yellow BM of the distal long bones (PFAS negative) (4, 5, 7). The integration of macroscopic characteristics and single-cell response at the tissue level resulted in the terms “regulated” BM adipose tissue (rBMAT) and “constitutive” BMAT (cBMAT), and prompted further investigation on their differential skeletal distribution, cell size, and relative lipid composition. Mechanistically, specific loss of rBMAT with conservation of cBMAT has been described in the long bones upon cold exposure, and in lipodystrophic *Ptrf* knock-out mice, while an overall loss of BMAds is characteristic of *c-kit* mutant *W/Wv* mice (5, 8, 9).

The association between increased vertebral fracture risk and BM adiposity (BMA) in humans, as well as hematopoietic stem cell (HSC) quiescence and BMA has prompted increased interest in marrow adiposity. Adipocytic conversion of the marrow has been associated to increased vertebral fracture risk in humans, although a cause-effect relationship is not clearly established (10). While mature BMAds generally induce HSC quiescence in humans and mice, BMAd precursors, and their adiponectin-expressing immature counterparts support hematopoietic expansion *in vitro* and *in vivo* (11–18). In mouse models and patients suffering acute BM failure or receiving chemo- or radiotherapy, a rapid and massive adipocytic infiltration of the BM takes place (“red-to-yellow” transition). Following treatment with intensive chemotherapy, and sometimes HSC transplantation, hematopoietic recovery ensues when BMAds recede and functional hematopoiesis is restored (“yellow-to-red” transition). In the clinic, hematopoietic activity is assessed by pathologists' scoring of hematopoietic cellularity in trephine bone biopsies of the iliac crest, and is functionally correlated with circulating blood cell counts (19). Hematopoietic cellularity assessment at low power examination is performed systematically for all diagnostic trephine biopsies, and complementary to immunohistochemistry molecular and cytogenetic techniques for establishing the diagnosis of certain hematological disorders characterized by either hypercellularity (e.g., myeloproliferative neoplasias) or hypocellularity (e.g., hypoplastic bone marrow failure syndromes), or to follow disease progression and response to treatment (e.g., myeloablation for acute leukemias).

*In vivo*, Gold Standard quantifications of BMA in the research setting are contrast-enhanced (CE) osmium tetroxide (OsO_4_) lipid staining coupled to micro-computerized tomography (μCT) for rodents, and magnetic resonance imaging (MRI) fat-to-water ratio for human. Although highly informative, especially on higher resolution such as nano-CT, this method is costly and sample preparation requires handling of highly toxic products. Thus, accessibility to CE μ/nanoCT and/or MRI expertise can be limited outside of clinical laboratories or specific research settings.

Given the context-dependent heterogeneity of BM compartments and the challenges associated to the accessibility and longitudinal reproducibility of Gold Standards, we set out to develop a tool that could provide quantitative information on both BMA and hematopoietic cellularity with means accessible to most laboratories. Histological information obtained from hematoxylin-and-eosin (H&E) stained sections has long been the standard method to provide information on the architecture of biological samples. While the BM is a particularly difficult organ for histological analysis, in part due to the soft and hard tissue that are juxtaposed, the overall analysis of its multiple components (bone, blood progenitors, adipocytes) provides important information to understand its physiopathology. Available histomorphometric analysis programs usually specialize in detecting individual components, and either require targeted staining protocols to detect specific components within the bone fraction, or use less adapted tools from the extramedullary adipose field to quantify the BMAd fraction [(8, 9, 20–27); and also reviewed in (6)]. In order to reduce inter-observer variability and to quantify sections of entire murine bones rather than representative fields of view, automation and standardization of methods are key. We have developed a semi-automated digital pathology plugin compatible with QuPath (28), named *MarrowQuant*, which subdivides and quantifies the total marrow area (Ma.Ar) in H&E stained mid-sections of mouse bones. The plugin subclassifies an user-defined area (“Tissue Boundaries,” see definitions under Image Processing Logic) into four compartments: the mineralized bone, the hematopoietic, adipocytic, and interstitial/microvascular compartments, as well as an unattributed area if present. Upon segmentation, *MarrowQuant* then provides a BMAd area (Ad.Ar) and size distribution for individual adipocytes, both in the context of BMAds and of extramedullary adipocytes. *MarrowQuant* thus provides absolute and relative areas for discrete BM compartments, calculating the hematopoietic cellularity and adiposity on H&E stained sections from murine whole-bone sections. Specifically, the results presented here validate the use of *MarrowQuant* in murine bone marrow in the context of aging as well as in longitudinal studies upon radiation-induced aplasia, and show compatibility with the analysis of human bone trephine biopsies. Additionally, the stand-alone *AdipoQuant* plugin quantifies relative number and performs adipocyte ghost size fragmentation on H&E-stained extramedullary adipose tissue. Adipocyte ghosts are the delipidated derivatives of adipocytes resulting from sample processing and resembling void areas on H&E.

Our software is thus complementary to other available plugins for BMA quantification [comparative review provided in (6)] in that, together with more classically reported values for individual adipocytes or bone components, it provides quantification of hematopoietic cellularity within the range of pathologists' evaluations, while providing overall architectural segmentation of the BM space. We expect *MarrowQuant* will be a valuable addition to the rapidly evolving Digital Pathology field for use in a fundamental research setting or as a tool for the “digitally-enhanced” pathologist in the clinical setting.

## Results

### Development of the Digital Plugin

**Figure 1:**
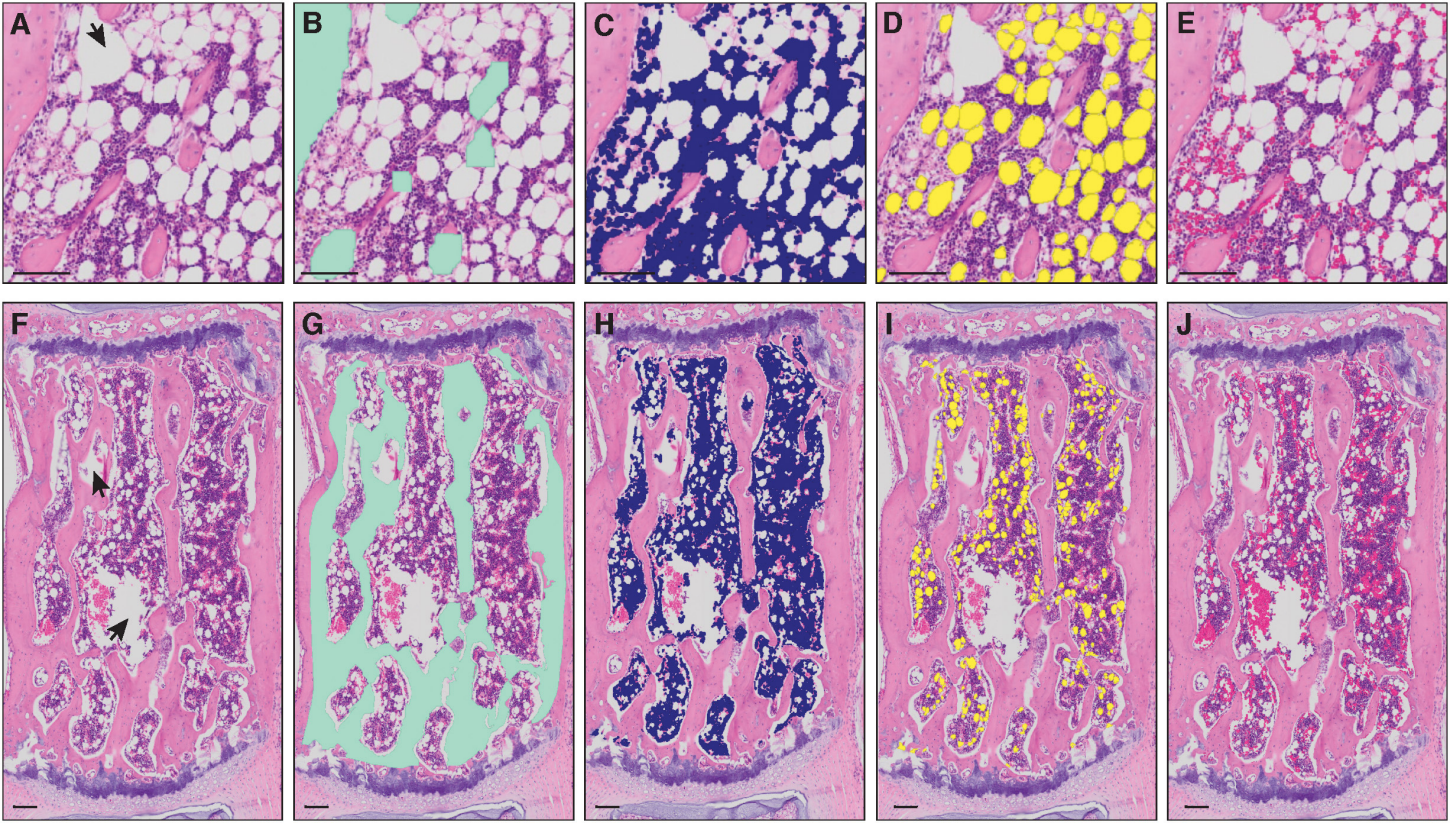
Masks of bone marrow compartment areas detected by *MarrowQuant*. **(A–E)** Proximal tibia and **(F–J)** caudal vertebra (Cd2). Arrowheads in **(A,F)** represent manually excluded *artefacts*. **(A,F)** Unprocessed H&E image, **(B,G)** bone detection (green), **(C,H)** nucleated cell detection (violet), **(D,I)** adipocyte ghost detection (yellow), **(E,J)** interstitium and microvasculature (pink). Image from the proximal tibia at day 15 post lethal irradiation and total bone marrow transplant **(A)** and caudal vertebra 2 **(F)** of a C57BL/6 2-months-old female mouse housed at room temperature fed a standard ad libitum chow diet. Scale bars are 50 μm **(A–E)** and 100 μm **(F–J)**. *MarrowQuant* user-defined parameters set at recommended values for bone marrow analysis (minimum adipocyte size: 120 μm^2^, maximum adipocyte size: 5,000 μm^2^, minimum circularity: 0.3, exclude edges: false).

**Figure 2:**
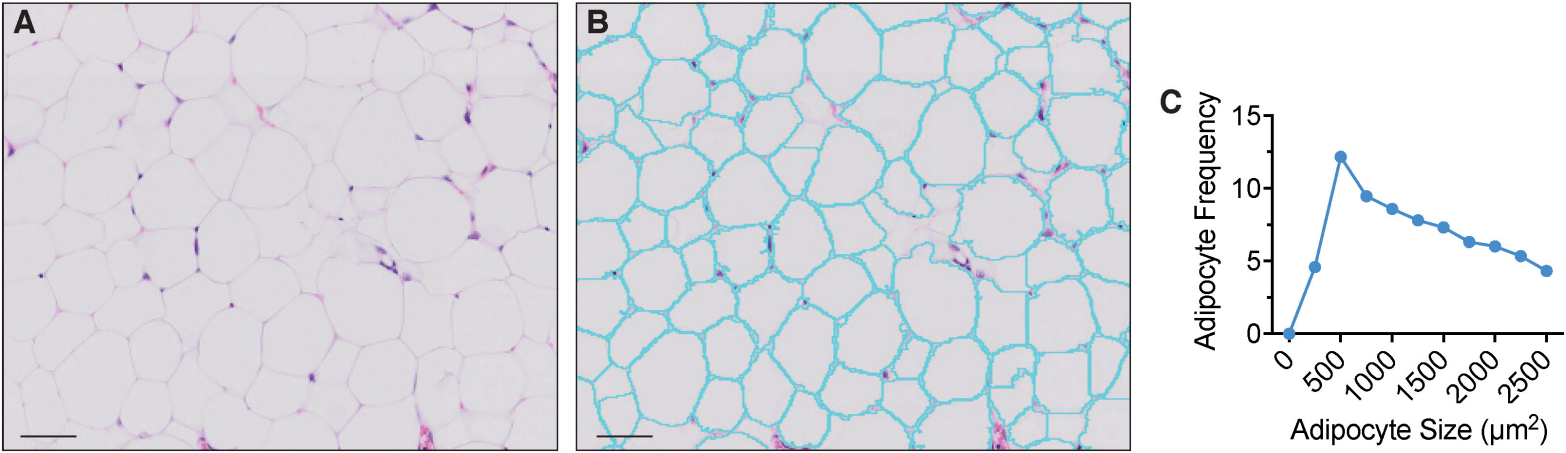
Extramedullary adipocyte fragmentation by *AdipoQuant*. **(A)** unprocessed image, **(B)** adipocyte ghost membrane detection and fragmentation (cyan), **(C)** adipocyte size fragmentation distribution. Omental adipose tissue from a C57BL/6 10-weeks-old female mice housed at room temperature fed a standard *ad libitum* chow diet. Scale bars are 50 μm. *AdipoQuant* user-defined parameters set at recommended values for extramedullary adipocytes (minimum adipocyte size: 300 μm^2^, maximwn adipocyte size: 2,500 μm^2^, minimum circularity: 0, exclude edges: true). Binning has been set to 250 μm^2^.

Based on a combination of color and texture of whole-bone H&E stained sections as compared to background, we found *MarrowQuant* to most reliably perform marrow quantification when set to segment bone sections into four mutually exclusive detected compartments (Figure 1): (i) cortical and trabecular bone, (ii) nucleated/hematopoietic cells, (iii) BMA, based on adipocyte ghost detection, and (iv) interstitium/microvasculature, which recognizes both red blood cells (RBCs) and the eosinophilic protein or serous infiltrate that fills the remaining marrow space. If present, areas within the marrow space that are not recognized as any of the above will be categorized and shown as “Undetected area.” The Ma.Ar is used as denominator (see “Image processing logic” for definitions) to calculate the percentage of each of the detected areas apart from percentage bone which the user may calculate themselves. Percentage of trabecular bone can be calculated if the tissue boundaries encompass only the marrow cavity, whereas percentage of cortical plus trabecular bone can be calculated if the tissue boundaries also include the cortical bone. From the adipocyte ghost segmentation, *MarrowQuant* additionally approximates the adipocyte count and size distribution. This function can also be applied to extramedullary adipose tissue sections with the stand-alone *AdipoQuant* function (Figure 2). The *MarrowQuant* plugin for bone sections operates through QuPath using Fiji as an extension, thus allowing the user to work with a more intuitive interface.

#### Summary of the Image Processing Logic

**Figure 3:**
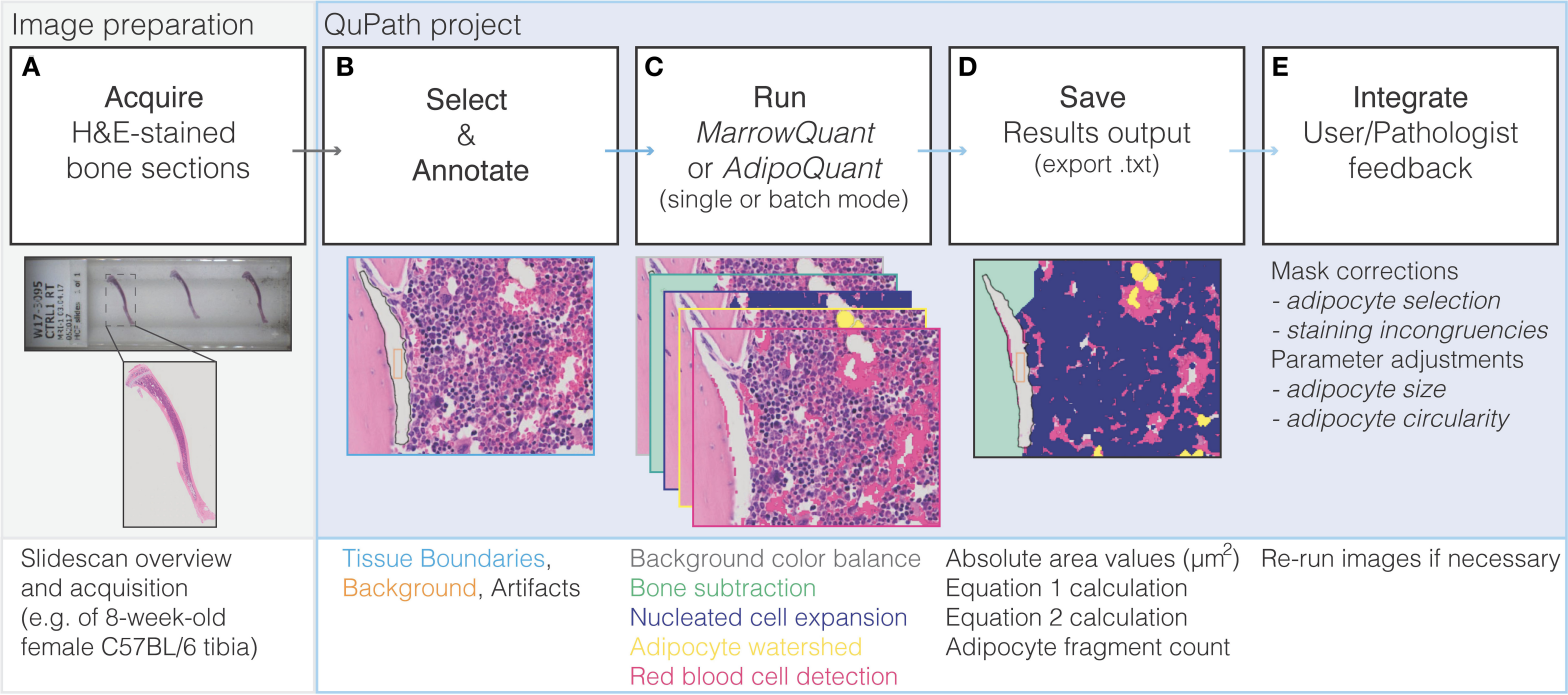
Flow diagram of *MarrowQuant* logic. **(A)** A slidescan is acquired of an H&E stained bone section and loaded into QuPath. **(B)** User defines Tissue Boundaries, Background, and Artifacts with the help of the magic wand tool. **(C)** *MarrowQuant* is run on the created qProject in single or batch mode with preset or user-adjusted parameters. **(D)** Results can be saved as bone marrow mask images and a.txt file of area and calculated outputs. **(E)** User and/or pathologists' feedback is integrated by removing false-positive adipocyte selection and if necessary, processing images with adjusted adipocyte parameters.

The workflow underlying the *MarrowQuant* processing is shown in Figure 3. Image files from total slide scans acquired at 20x magnification (VSI format or extracted TIFF files) are directly loaded into QuPath for processing (Figure 3A). Firstly, and prior to image processing, the user must manually identify the region of interest (ROI) to be analyzed, denoted as “Tissue Boundaries” (Figure 3B). The wand tool in QuPath greatly simplifies this step. The user may also classify at this step regions that should be excluded from processing as “Artifacts.” In our dataset, we excluded significant fixation artifacts at this step, which are common upon retraction of the marrow tissue from the endosteum. For our dataset, we also systematically excluded the retraction artifact that creates a central vein lumen. Secondly, a representative small area within the Tissue Boundaries must be defined as “Background” and will serve as a reference background correction factor for the whole ROI. These regions are selected manually by the user with built-in drawing tools in QuPath and assigned the corresponding annotation class (see *MarrowQuant* Demo in Supplementary Video 1, Supplementary Datasheets 1 and 2). Preset parameters for adipocyte ghost circularity, roundness, and size are defined, but may be changed by the user depending on their application (e.g., tissues of different origin). For our dataset, we kept these parameters conservative to include all possible adipocytes and thereby test the robustness of *MarrowQuant* (see Methods and Figures 1, 2 legends for details). More restrictive values can be set and should be determined by the user on their particular dataset.

#### Extramedullary Adipocyte Quantification With *AdipoQuant*

As suggested by the opposing effects of extramedullary adipocyte hyperplasia and adipocyte hypertrophy, the measurement of adipocyte size and number has become relevant in the context of multiple patho/physiological conditions (29, 30). We thus developed *AdipoQuant* to provide the adipocyte detection function as a stand-alone plugin which detects the total area covered by adipocyte ghosts and identifies individual adipocyte ghosts through recognition of the remaining membranes, providing a fragmentation size distribution count which can be applied to extramedullary white adipose tissues (Figure 2). Of note, adipocytes can only be identified as lipid ghosts in H&E images, as the lipid content in the vacuoles is dissolved on the alcohols used for the processing. Changes in relative adipocyte size of a whole adipose tissue may be thus quantified with the *AdipoQuant* script directly. The user must manually outline “Tissue Boundaries,” unwanted “Artifacts,” and the “Background” as described above. Not drawing any “Tissue Boundaries” will result in the entire image being analyzed. A.txt file is generated upon processing which lists the adipocyte ghost fragmentation count and size. The tool relies on identification of intact, clearly-stained H&E membranes for the most reliable generation of a watershed algorithm that fragments the mask. A mask is a pixel annotation of a specific region of the original image resulting from image processing (31). Therefore, a reliable size distribution is highly dependent on the quality of sample preparation (including staining, sectioning, and image acquisition).

#### Bone Marrow Quantification With *MarrowQuant*

Following the initial manual steps performed by the user in setting “Tissue Boundaries,” excluding “Artifacts,” and defining the “Background” within the QuPath environment as described above (Figure 3B), the *MarrowQuant* plugin first identifies the bone compartment within the given “Tissue Boundaries.” The bone mask is then subtracted to generate the total area of interest that we define as the “Total Marrow Area” (Ma.Ar) ascribing to the nomenclature recommendations for BMA (32). Within the “Total Marrow Area,” nucleated cells are detected to generate the “Hematopoietic Cellularity” mask. A watershed mask then detects and defines adipocyte ghosts by their intact cell membranes in the remaining area to generate the “Adiposity” mask. Finally, both the serous infiltrate and the RBCs, located either within the microvasculature or dispersed across the marrow space, are detected based on their eosinophilic properties to define the “Interstitium/microvasculature” mask. The remaining area within the “Total Marrow Area,” if any, is then quantified as unattributed “Undetected Area.” The five masks are mutually exclusive in the priority order specified above (Figure 3C). Therefore, “Total Marrow Area” (Ma.Ar) constitutes the sum of “Hematopoietic Cellularity” plus “Adiposity” plus “Interstitium/microvasculature” plus the unattributed “Undetected Area.” Derived parameters are the adipocyte size fragmentation distribution, and the ratio of hematopoietic area over the hematopoietic and adipocytic areas combined (see Equations 1 and 2 below). A mask output for the four defined compartments overlaid on the original image lets the user confirm the results (Figures 3D,E).

### Validation of *MarrowQuant* With Gold Standards

μCT is an established technique used to quantify changes in bone mineral density and bone architecture. It is now widely used in the field of BMA for lipid detection. *Ex vivo* CT imaging of the native bone is combined with a second CT acquisition after bones have been decalcified and stained with the highly lipophilic dye OsO_4_. This procedure is usually referred to as OsO_4_-contrast-enhanced μCT or OsO_4_-CE-μCT. The ground truth assessment of hematopoiesis constitutes pathologists' assessment of H&E stained paraffin- or plastic-embedded bone sections. We therefore compared these Gold Standard evaluations of murine marrow, first for BM adiposity then for hematopoietic cellularity, to *MarrowQuant* detection. Importantly, mid-bone sections were defined by a complete bone silhouette plus the presence of the distal marrow space in the mid-sagittal tibial sections, and the femoral head marrow space in mid-longitudinal sections of the femur.

#### BM Adipose Quantification

**Figure 4:**
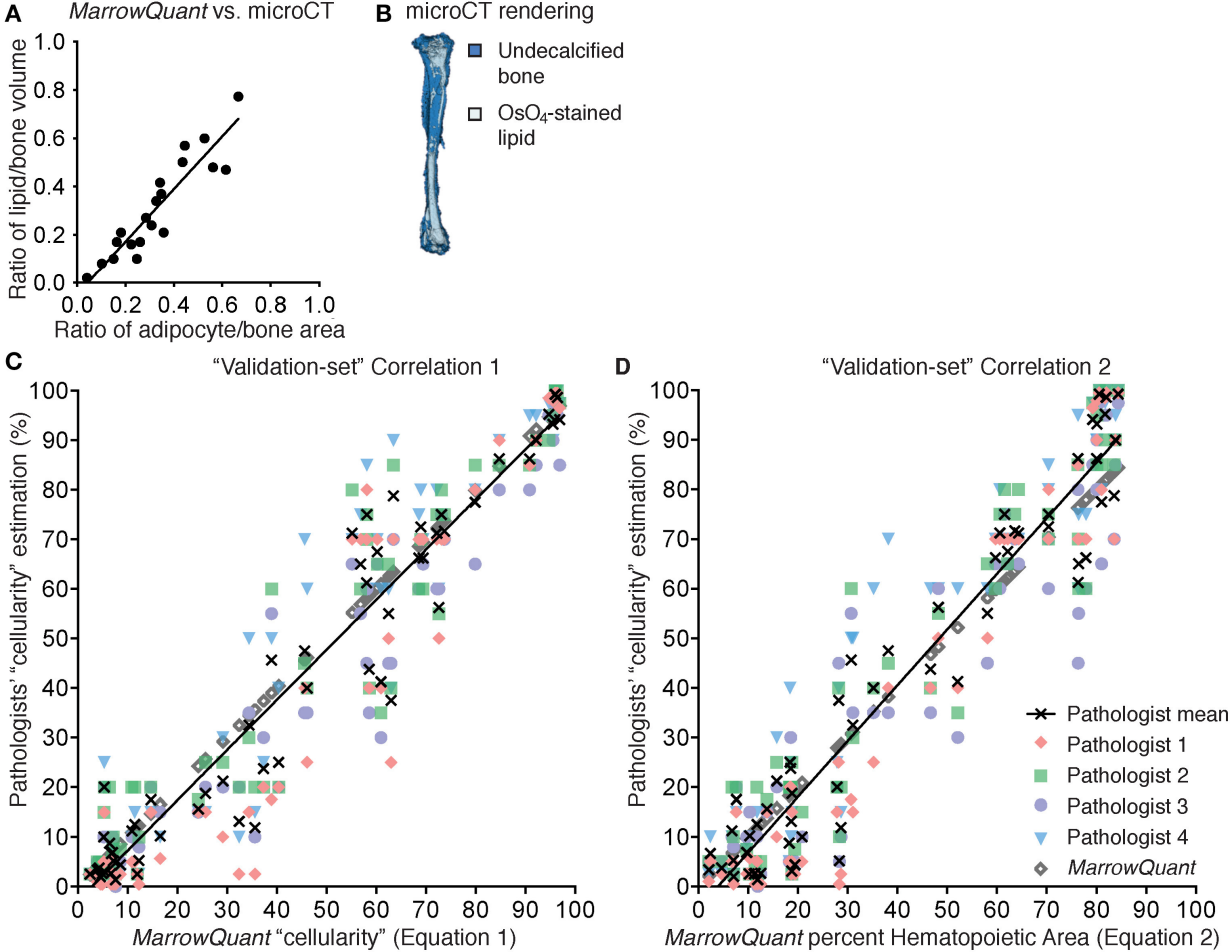
Correlation to gold standards for *MarrowQuant* bone marrow adiposity and hematopoietic cellularity measurements. **(A)** *MarrowQuant* adipose area vs. osmium-tetroxide contrast-enhanced CT measurements of lipid (*n* = 23 bones, *R*^2^ = 0.84). Quantifications performed on femurs and tibiae of 18-months-old male mice, 8-weeks, 9-months, or 18-months-old female mice and of a select ion from **(C)**. **(B)** μCT rendering (white: osmium-tetroxide stained lipids; blue: undecalcified bone) of a tibia from an 18-months-old C57BL/6 female mouse. **(C)** *MarrowQuant* “cellularity” (Equation 1) vs. Pathologists' “cellularity” assessment on the “validation-set” of images of full-length bones (Pathologists *n* = 4, images *n* = 59, *R*^2^ = 0.93). **(D)** *MarrowQuant* percent Hematopoietic Area (Equation 2) vs. Pathologists' “cellularity” assessment of the “validation-set” (Pathologists *n* = 4, images *n* = 59, *R*^2^ = 0.93). Evaluations done for femurs and tibiae of 2-months-old female mice at 0–42 days post lethal irradiation and transplant of 125,000 total bone marrow cells. All mice were C57BL/6 housed at room temperature and fed a standard ad libitum chow diet. OsO4, osmium tetroxide; μCT, micro-computerized tomography.

We first compared the volumetric assessment of lipid content via OsO_4_-CE-μCT in C57BL/6 murine long bones to the *MarrowQuant* two-dimensional assessment of BM adipose area in mid-bone sections. Quantification of contralateral bones (right vs. left) with the two techniques over a range of percentage adiposity spanning from 0 to 64% indicates a high correlation (Figure 4A, *n* = 23, *R*^2^ = 0.84) despite the comparison of a volumetric (Figure 4B) with a two-dimensional assessment. In our dataset, 2D quantification of mid-sagittal sections are thus a robust estimate of volumetric renderings of bone and adiposity in the contralateral bone.

#### BM Hematopoietic Quantification

In spite of the species-specific differences discussed later, we decided to compare the performance of *MarrowQuant* with pathologists' assessment of hematopoietic cellularity in murine bones. To set up the *MarrowQuant* code and optimize the model parameters we curated a “training-set” of 89 images from murine bones at homeostasis or post-irradiation aplasia, spanning pathologist evaluated hematopoietic cellularity from 0 to 100%.

In human pathology, hematopoietic “cellularity” is defined as “the proportion of cellular elements relative to marrow adipose tissue” (33, 34), and therefore uses the sum of the hematopoietic and adipocytic areas as denominator for the calculation of “cellularity” (Equation 1, % hematopoietic cellularity). The pathologist definition of hematopoietic “cellularity” underscores the reciprocal relationship between the hematopoietic and adipose tissue in the BM.

(1) $$\begin{array}{r} ”Cellularity”=\frac{hematopoietic\;area}{\left(hematopoietic+adipocytic\right)\;area}\; \end{array}$$

We found a strong linear correlation between the *MarrowQuant* calculation of hematopoietic cellularity and the mean of the pathologist's scoring of “cellularity” (Equation 1, Figure S1A, *n* = 89, *R*^2^ = 0.98). *MarrowQuant* “cellularity” values thus fell within the inter-observer variability of four independent pathologists' assessments, with a higher correlation to the mean at high “cellularity” values. *MarrowQuant* performance at estimating “cellularity” was subsequently confirmed on an independent “validation-set” from 59 images of murine long bones (Figure 4C, *n* = 59, *R*^2^ = 0.93).

In accordance with the *MarrowQuant* logic to subclassify the marrow space into three different compartments (hematopoietic, adipocytic, and interstitium/microvasculature) and an unattributed “undetected area” when present, we then calculated the hematopoietic cellularity as defined by hematopoietic area over total Ma.Ar (Equation 2, % Hematopoietic Area).

(2) $$\begin{array}{r} \%\;Hematopoietic\;Area=\frac{hematopoietic\;area}{total\;marorw\;area}\; \end{array}$$

We found that *MarrowQuant* measurement of percent Hematopoietic Area (Equation 2) progressed linearly when compared to the mean of the pathologists' “cellularity” scoring (Equation 1). Equation 2, however, better correlates to the mean of the pathologists' “cellularity” scoring at low hematopoietic content (Figure S1B, *n* = 79, *R*^2^ = 0.96). The *MarrowQuant* measurement of percentage Hematopoietic Area was also validated on an independent “validation-set” (Figure 4D, *n* = 59, *R*^2^ = 0.93). Note that *MarrowQuant* evaluation of percent Hematopoietic Area does not reach 100%, as opposed to pathologist-rated “cellularity,” precisely because it uses a different denominator that takes into account the entirety of the marrow space including the interstitium/microvasculature compartment.

In conclusion, in C57BL/6 mice, *MarrowQuant* 2D evaluation of BM adiposity (% Adipocytic Area/ Total Ma.Ar) in mid-bone sections highly correlated with gold standard OsO_4_-CE-μCT detection of marrow lipids in 3D measurements. Evaluation of hematopoietic content with *MarrowQuant* also highly correlated with gold standard “cellularity” scoring by pathologists. For evaluation of the hematopoietic compartment, *MarrowQuant* generates results according to two different equations that differ on the denominator. Equation 1 better correlates to the mean of the pathologist scoring in samples of high cellularity, while Equation 1 better correlates at low cellularities. For *MarrowQuant* evaluation of the percentage Hematopoietic Area as compared to other BM compartments, only Equation 2 is applicable as *MarrowQuant* uses Total Marrow Area as a denominator for all compartments.

### Application of *MarrowQuant*

We hypothesized that quantification of BM components as a first architectural assessment of BM regeneration may provide valuable information on homeostatic states and disease progression. The C57BL/6 mouse constitutes the most widely used animal model in experimental hematopoiesis. Its skeleton is often assumed to be homogeneously hematopoietic, whereas it has now been well-described that BMAds are interspersed within specific regions of the marrow (5, 17).

#### Skeleton of the Homeostatic C57BL/6 Female Mouse

**Figure 5:**
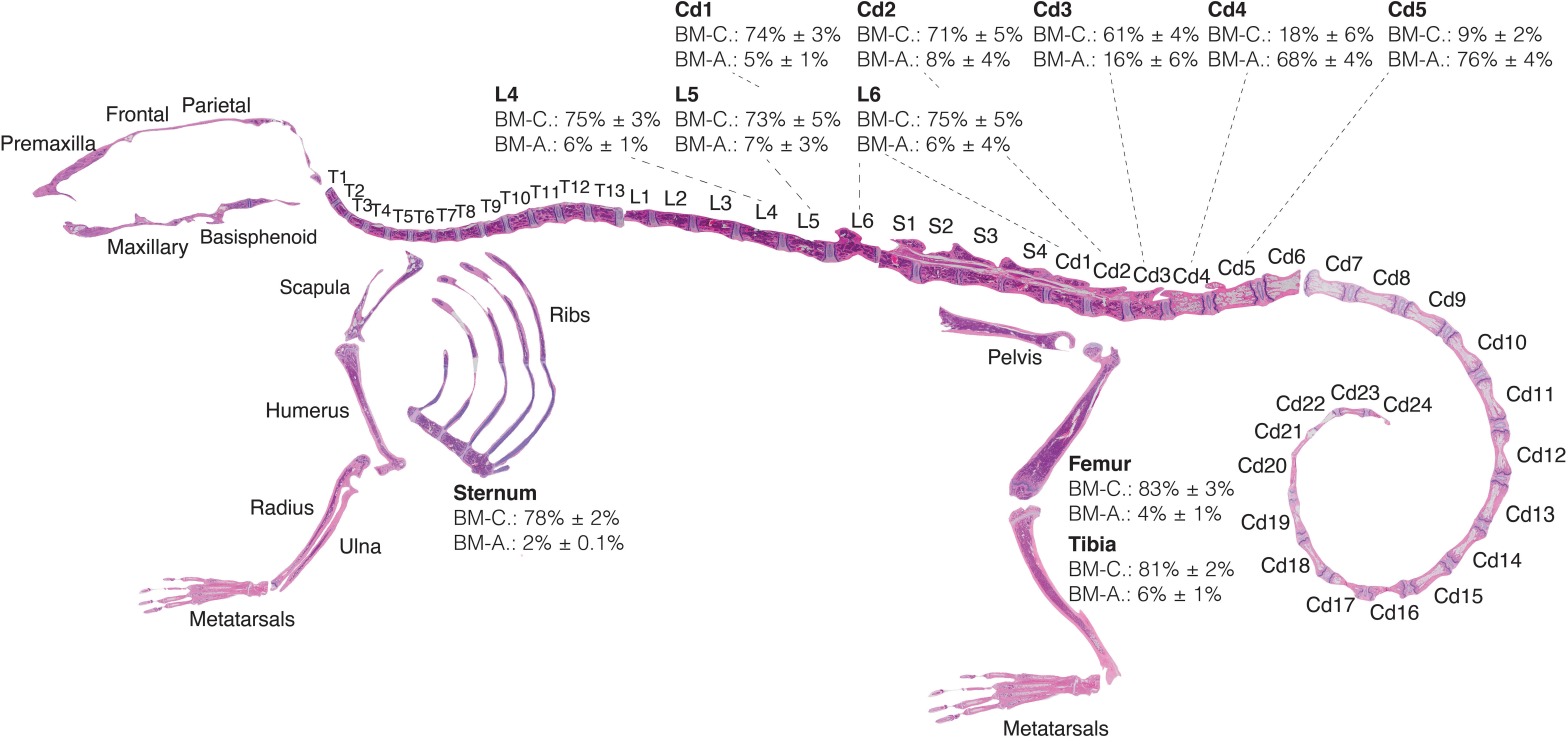
Red-to-yellow marrow transition in homeostasis. Eight-weeks old C57BL/6 reconstructed skeletal map locates the proximal-to-distal red-to-yellow marrow transition in the first caudal vertebrae and proximal-to-distal tibia approximately at the site of the fibular insertion. Skeletal map and quantifications performed for bones of female mice housed at room temperature and fed a standard *ad libitum* chow diet. Percentages of MarrowQuant cellularity (BM-C) and adiposity (BM-A) from Equation 2 are shown for select bones (femur, *n* = 6; tibia, *n* = 6; sternum, *n* = 10; lumbar, *n* = 7; tail, *n* = 6; all are mean ± s.d.). Cd, caudal (vertebrae); L, lumbar; S, sacral; T, thoracic.

**Figure 6:**
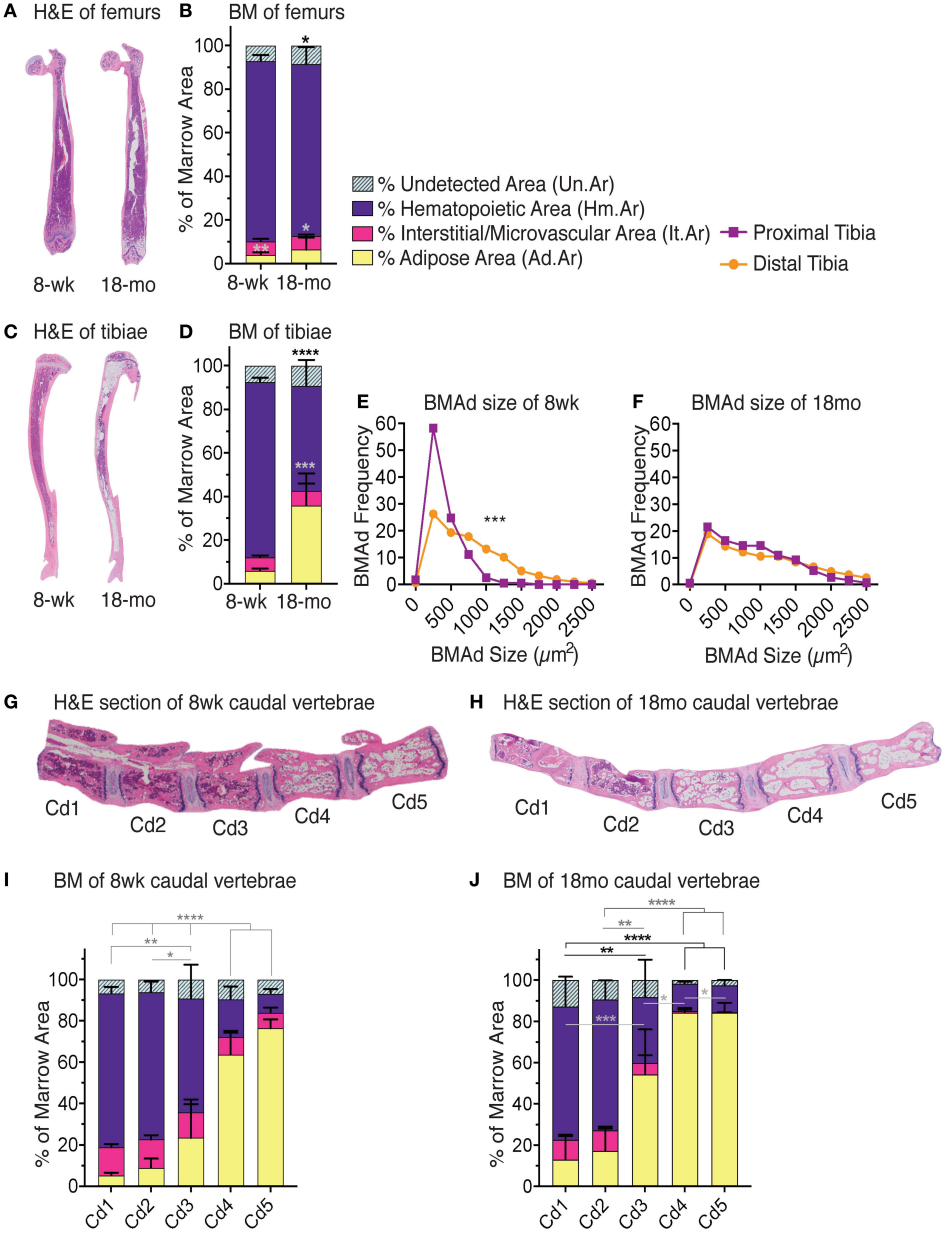
Red-to-yellow transition with aging. Representative H&E images of femurs **(A)** tibiae **(C)**. Note high adiposity on proximal and distal but not mid-shaft of 18-months-old female tibia. *MarrowQuant* analysis for **(B)** femurs (*n* = 3) and **(D)** tibiae (*n* = 3) from 8-weeks-old and 18-months-old C57BL/6 females. BM adiposity is significantly higher in 8-weeks-old tibias vs. femurs (*P* = 0.0045) and in 18-months-old tibias vs. femurs (*P* = 0.0316). Hematopoiesis is greater in 18-months-old femurs than tibias (*P* = 0.0993). Error bars represent mean ± s.d. *P*-values are indicated for hematopoietic compartment (purple) and adipocytic compartment (yellow) are ^*^P < 0.05, ^**^P < 0.01, ^***^P < 0.001, ^****^P < 0.0001 by Student's *t*-test for cellularity (black) and adiposity (gray). **(E)** BMAd fragmentation distribution of the proximal (purple) and distal (orange) 8-weeks-old tibia (*n* = 10) vs. **(F)** the 18-months-old tibia (*n* = 3). ^***^P < 0.001 by multiple comparisons test. Representative mid-longitudinal H&E section of bone marrow compartments in the proximal-to-distal caudal vertebrae of **(G)** 8-weeks-old and **(H)** 18-months-old mice. *MarrowQuant* analysis of caudal vertebrae in **(I)** 8-weeks-old (*n* = 6) and **(J)** 18-months-old (*n* = 4) female mice. ^****^P < 0.0001, ^**^P < 0.01 for cellularity and adiposity and ^*^P < 0.05 for cellularity by two-way ANOVA. Mice were C57BL/6 females housed at room temperature and fed a standard ad libitum chow diet. BM, bone marrow; BMAd, bone marrow adipocyte; H&E, hematoxylin and eosin; mo, month; wk, week.

Here we provide for the first time a quantitative BMA skeletal map of the homeostatic C57BL/6 8-weeks-old female mouse, which reveals a transition of low adiposity, red BM (high cellularity) to high adiposity, yellow BM (low cellularity) in the proximal to distal regions of the skeleton (Figure 5). This transition is most notable in the proximal-to-distal tibia (Figure 6C), and highly predictable in the caudal vertebrae of the tail at the level of the Cd3–Cd4 transition (Figures 5G,H). The extremities of the skeleton such as the metatarsals or caudal tail vertebrae (e.g., Cd5, 76 ± 4% adiposity) are highly adipocytic whereas the axial skeleton such as the sternum (2.0 ± 0.1% adiposity) and thoracic/lumbar vertebrae (e.g., L4, 6 ± 1% adiposity) are almost entirely hematopoietic. These gradients of red and yellow marrow have also been reported in human, where the adult distal skeleton is nearly completely adipocytic (3, 35). Location-specific BMA may be explained from the perspective of skeletal development, musculoskeletal forces, and temperature gradients (36).

#### Long Bones of the 18-Months-Old C57BL76 Female Mouse

BMA has been described to increase with age, which may in part be attributed to the terminal differentiation and depletion of BMAd precursors, and in part to the potential protective function assigned to mature BMAds of surrounding cells such as low-proliferating HSCs. In the clinical diagnostic setting, values for normal cellularity of standard trephine biopsies from the pelvic bone in humans are interpreted according to age such that normal, age-adjusted cellularity is described by the formula: normal cellularity = 100% – age (years) ± 20% (33).

In C57BL/6 mice, significant differences in BMA are seen between 8 weeks and 18 months of age (Figure 6). The femurs of juvenile mice at 8 weeks of age contain relatively few BMAds (2 ± 1%) that are mostly located at the distal site. With age, BMA in the femur of female C57BL/6 mice increases slightly to 4 ± 2% adiposity (*P* = 0.03) and also appears in the proximal femur (Figures 6A,B). While the distal tibia already contains BMAds at an early age with 4 ± 1% adiposity at 8 weeks old, BMA expands further up the BM cavity, especially at the epiphysis of the proximal tibia to 31 ± 14% (*P* = 0.01) at 18 months old (Figures 6C,D). BMAd size fragmentation distributions tibia in homeostasis (8-weeks-old C57BL/6 females) shows the distribution shifted toward larger adipocyte size in the distal tibia as compared to the proximal tibia (Figure 6E) as has been previously reported (5). Adipocyte size fragmentation in the tibiae of aged mice (18-months-old C57BL/6 females) show comparatively larger adipocytes as compared to the young animals (Figure 6F). Interestingly, older mice present no difference in the size distribution of distal vs. proximal adipocytes across the tibia, suggesting reduced remodeling as compared to young mice (Figures 6E,F, compare orange lines). BMA is also increased in the proximal tail of aged mice while maintaining the red-to-yellow marrow transition in the first caudal vertebrae (Figures 6G,H). Other strains such as the C3H, with high bone mass, or NSG, that are prone to obesity, have higher BMA than the C57BL/6 strain (not shown).

#### Irradiation-Induced BM Aplasia in the Skeleton of the C57BL/6 Female Mouse

**Figure 7:**
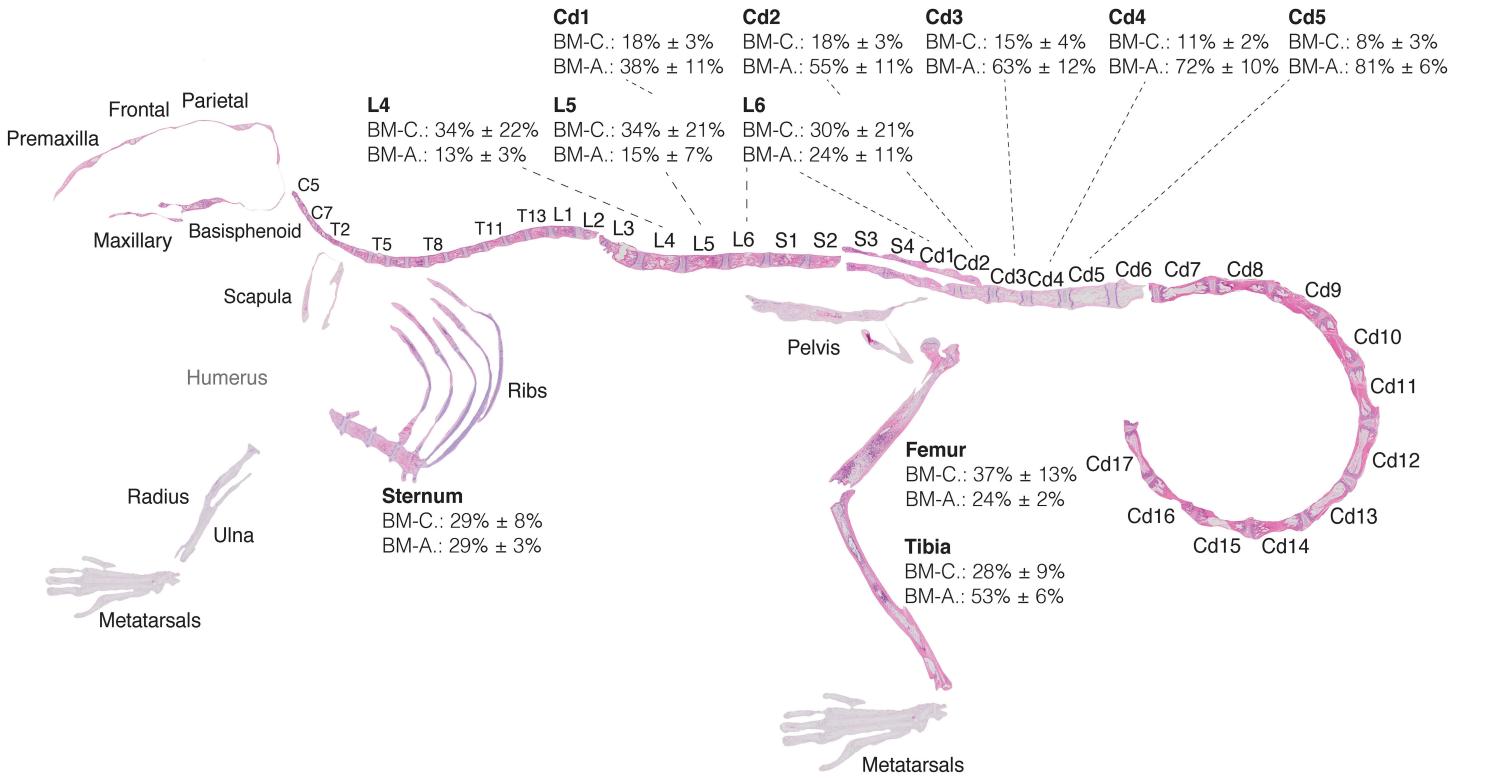
Red-to-yellow-to-red transition in bone marrow aplasia and hematopoietic recovery. Skeletal map of an 8-weeks-old C57BI/6 female mouse 17 days post-lethal irradiation and transplant of 125,000 total bone marrow cells. Results for *MarrowQuant* quantification of hematopietic cellularity and adiposity are shown for select bones (femur, *n* = 3; tibia, *n* = 10; sternum, *n* = 3; lumbar, *n* = 4; tail, *n* = 4, all error bars represent mean ± s.d). Note that average adiposity (BM-A) and hematopoietic cellularity (BM-C) are homogenous across the axial skeleton (lumbar vertebrae and sternum) and femur, while the tibia and caudal vertebrae present higher adiposity and lower cellularity. BM-C is higher in sternum than Cd5 (*P* = 0.0001) and Cd4 (*P* = 0.0002), while BM-A is higher in Cd-5 (*P* < 0.0001) and Cd4 (*P* = 0.0002) than sternum by Student's *t*-test. Cd, caudal (vertebrae); L, lumbar; S, sacral; T, thoracic.

**Figure 8:**
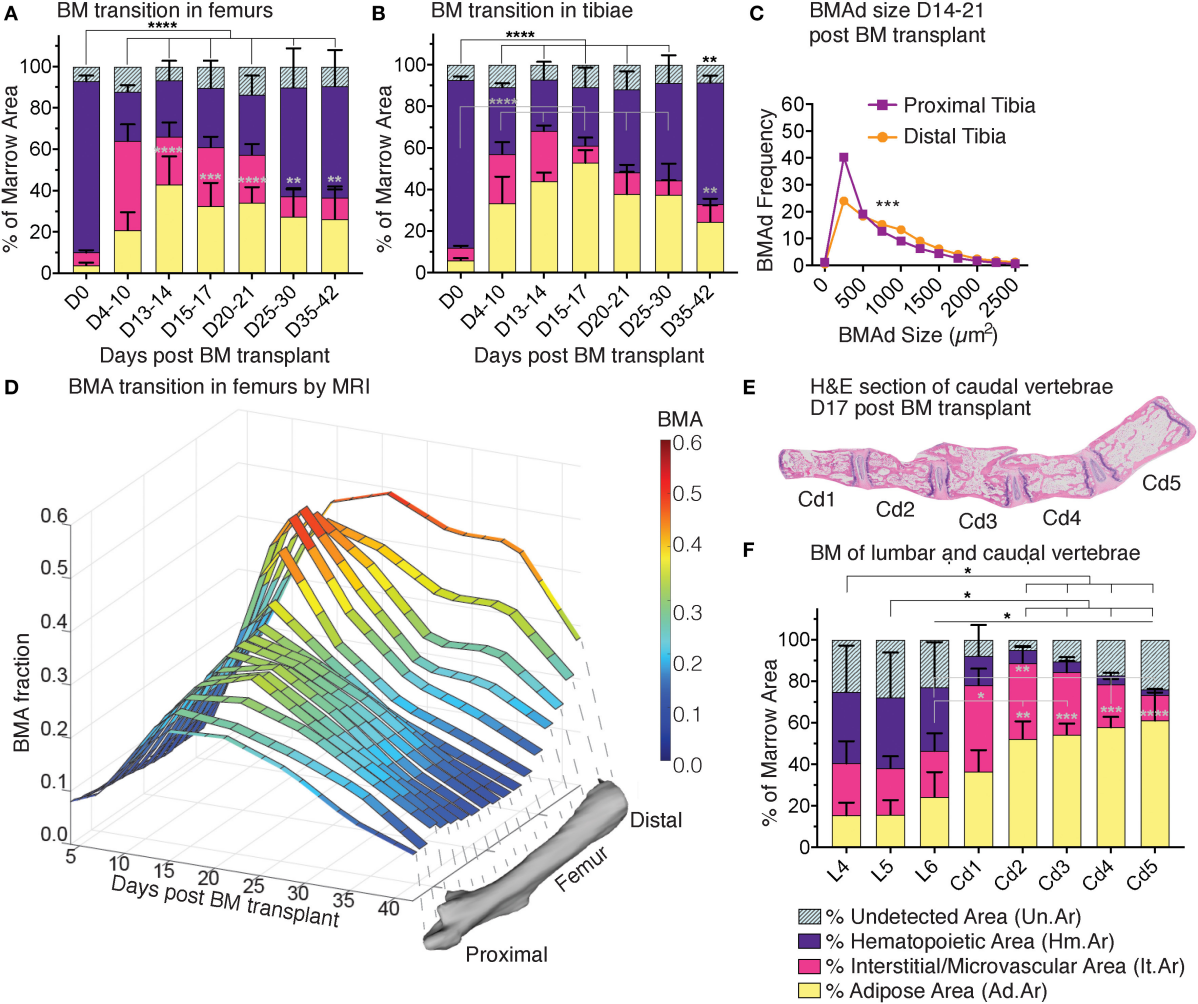
Red-to-yellow-to-red transit ion in bone marrow aplasia and hematopoietic recovery. *MarrowQuant* results for quantifications of **(A)** femurs and **(B)** tibiae in hematopoietic recovery at 0–42 days post lethal irradiation and transplant of 125,000 total bone marrow cells in 2-months-old C57BL/6 female mice. ^****^P < 0.0001, ^***^P < 0.001, ^**^P < 0.01, ^*^P < 0.05 by two-way ANOVA for cellularity (black) and adiposity (gray). **(C)** BMAd size fragmentation distribution in the proximal and distal tibia at peak of aplasia on days 17–21 (*n* = 10 tibiae) from *MarrowQuant* measurements extracted from **(B)**. **(D)** Changes in BMA fraction measured by MRI after lethal irradiation and total BM transplant as a time-course of recovery in the whole femur *in vivo* under the same conditions as **(A)**, *n* = 3–5 bones per time point. ^***^P < 0.001 by multiple comparisons test. **(E)** Mid-longitudinal H&E section and **(F)** quantification of bone marrow compartments in the proximal-to-distal caudal vertebrae at day 17 post lethal irradiation and transplant of 125,000 total bone marrow cells in 2-months-old C57BL/6 female mice. ^****^P < 0.0001, ^**^P < 0.01, and ^*^P < 0.05 for BM adiposity by two-way ANOVA. All error bars represent mean ± s.d. Mice were housed at room temperature and fed a standard *ad libitum* chow diet. BM, bone marrow; BMA, bone marrow adiposity; BMAd, bone marrow adipocyte; Cd, caudal (vertebrae); D, day; L, lumbar; MRI, magnetic resonance imaging; S, sacral; T, thoracic.

In certain pathophysiological circumstances such as in osteoporosis, anorexia, or obesity, the marrow becomes more adipocytic. In stress hematopoiesis, such as after chemo/radiotherapy to overcome malignant hematopoiesis, the plasticity of the marrow becomes very apparent. In the timespan of several weeks, the marrow undergoes a rapid “red-to-yellow” conversion, and then recovers thanks to residual or transplanted HSCs. We set up a murine stress hematopoiesis model by applying lethal irradiation immediately followed (day 1) by transplantation of a limited number of healthy BM cells. In this model, the marrow initially becomes necrotic and highly hemorrhagic due to the breakage of the marrow-blood barrier (day 4–10 post-transplant), such that the marrow space is characterized by a high content of RBCs and serous infiltrate. During this time, circulating white blood cells and platelets are reduced 10-fold (Figure S2). Shortly thereafter BMAds begin to occupy the marrow space such that the BM reaches peak adiposity at days 13–17 post-transplant in the femur and tibia (Figures 7, 8A,B). At this time, and in parallel to revascularization, the new hematopoietic system starts to recover. Around day 25 post-transplant, circulating blood levels show exit from severe thrombo- and neutropenia (<200 and <0.5 cells/mm^3^, respectively) (Figure S2). Over the next several weeks, blood counts increase and eventually return to normal levels of functional hematopoiesis. Importantly, the overall peak of aplasia and time of recovery in this model closely resembles that observed in patients undergoing HSC transplantation (37, 38).

Examination of the C57BL/6 skeleton at murine peak of aplasia shows that myeloablation renders the entire skeleton of the C57BL/6 8-weeks-old female mouse highly adipocytic and aplastic (Figure 7). Specifically, we find at day 17 post-irradiation a low hematopoietic cellularity (average BM-C 15–37%) in skeletal sites that are highly hematopoietic at homeostasis (average BM-C >50% hematopoietic cellularity, e.g., axial skeleton including sternum and vertebrae Cd1-3) (Figures 5, 7). Site-specificity variations including a proximal to distal hematopoietic gradient are conserved, and can be reliably measured in the lumbar to caudal transition (Figure 8F). Moreover, longitudinal analysis of the “red-to-yellow-to-red” transition in femurs (Figure 8A) and tibiae (Figure 8B) also indicated a peak of adiposity at days 14–17 post-irradiation, and a higher peak of adiposity in the tibiae than in the femurs.

The first 2 weeks post-irradiation entail important vascular remodeling. Quantitatively, the interstitium/microvasculature even constitute the most important compartment in some of the proximal sites (e.g., femur D4-10, Cd1 on day 17). Interestingly, when samples of varying cellularities post-irradiation are compounded (all images from Figure 4D), the measure of hematopoietic cellularity presents preferential reciprocity with the adiposity and interstitium/microvasculature compartments (Figure S3), suggesting that hematopoietic expansion or retraction might be the driver of marrow remodeling. Changes in BMAd size fragmentation of lethally irradiated mice as compared to homeostatic C57BL/6 8-weeks-old females, indicate a shift toward more and larger BMAds in the proximal tibia (Figures 6E, 8C, compare purple lines). The increase in number and size signifies hyperplasia and hypertrophy of the labile BMAds in the proximal tibia at the peak of aplasia putatively originating from a differentiation of skeletal stromal/stem cells or pre-BMAds. However, a small change in size fragmentation in the distal tibia, which is predominantly composed of stable adipocytes, indicates that constitutive BMA could also be undergoing some degree of remodeling. A similar “red-to-yellow-to-red” conversion occurs in the femur during the time of aplasia.

Just as with CE-μCT in homeostasis, we set to validate our findings with MRI as an available tool for Gold Standard measurements of BMA. BMA quantification performed in murine limb bones was possible through access to a 9.4 Tesla magnet for small animal imaging. We chose MRI *in vivo* imaging as an alternative to *ex vivo* imaging because repetitive imaging allows for a very significant reduction on the number of animals required to study the kinetics of marrow recovery, as compared to the *ex vivo* terminal experiments. MRI analysis validated the time course of marrow recovery in the femurs of mice undergoing lethal irradiation followed by BM transplantation, as compared to *MarrowQuant* analysis of full bone mid-longitudinal sections (Figure 8D). Additionally, MRI measurements also revealed that the proximal femur appears to recover more rapidly (max at D10 and decreases, Figure 8D) than the distal portion (max D15–D30, Figure 8D), which already contains some BMAds during homeostasis (stable/constitutive BMAds). In conclusion, volumetric measurements by MRI show a progression of fat signal that corresponds to the bidimensional quantification of adipocyte ghosts observed on histology with one of the most commonly used techniques to estimate fat content of a tissue *in vivo* (39).

#### Human Trephine Biopsies

*MarrowQuant* has been developed for quantification of mouse bone H&E stained paraffin sections and is also able to detect BM compartments in human trephine biopsies (Figure S4). While samples analyzed from mice have the entire bone encompassing the marrow still intact, trephine biopsies although similar in size (roughly 2 cm long and 2 mm in diameter) also have trabecular bone but not the cortical bone encompassing the marrow. Cells are larger in general (40) thus presenting a different texture to stained mouse sections and may also present with a larger degree of serous infiltrate that is not detectable with the current version of *MarrowQuant*. Additionally, due to their surgical nature, trephine biopsies are by definition hemorrhagic with RBCs detected throughout the majority of the interstitium. Note that currently *MarrowQuant* cannot separate the interstitial and the microvascular components. Despite these differences, initial correlations, indicate promising results (pathologists *n* = 2, images *n* = 32, *R*^2^ = 0.85, Figure S4F).

## Discussion

*MarrowQuant* provides a means to quantitatively evaluate overall BM architecture in H&E-stained sections in single- or batch-mode by subclassification into the bone, adipose, hematopoietic, and interstitial/microvascular compartments. Systematic comparison of training and validation sets with gold standards for adiposity and hematopoietic cellularity showed robust correlations. To our knowledge, this is the first semi-automated digital pathology tool to quantitatively evaluate murine marrow composition and in particular the hematopoietic and interstitial/microvascular compartment. Other approaches in murine and human tissues have been described which focus mainly on bone morphometry and use stains non-compatible with concurrent hematopoietic evaluation such as Goldner's Trichrome or Von Kossa or TRAcP (6, 41).

While standard practices for evaluating bone morphometry and hematopoietic cellularity have existed for many years, the field of BMA is newly emerging and thus methods of quantification are currently being defined. Automatization of sample preparation and quantification is required to increase accuracy, comparability, and reproducibility of results (6). This is rapidly developing in clinical and experimental pathophysiology with image-analysis programs emerging for quantification and detection of specific cell types related to disease progression (42, 43). Indeed, the high-quality image collection of over 192 murine H&E-stained bone sections that composes our dataset may become useful in training deep-learning algorithms for full automatization and finer detection of BM compartments. Histomorphometry reveals abundant architectural information about a specimen that may be missed by the gating inherent to immunofluorescence. In the future, highly multiplexed immunofluorescence staining has the possibility to reveal as much information of cellular composition as conventional flow cytometry analysis while conserving information on tissue structure (44). *MarrowQuant* constitutes an example of a histomorphometry digital pathology tool that can provide quantitative data complementary to visual analysis of architectural features.

### BM Compartment Detection

*MarrowQuant* relies on the user's definition of a region of interest and the exclusion of artifacts that may arise from sample processing (such as fixation retractions). High-quality stained sections provide best results as the tool relies on intact adipocyte membranes and an even staining coloration for quantification. Image acquisition is also an important initial aspect of preparation as the tool relies on background identification, and therefore stitched images should not show a gradient that would produce an uneven shade of the background and influence subsequent processing steps. Despite these measures that must be accounted for during setup, the automation of image quantification with *MarrowQuant* means that numerous samples can be batch processed. While the automated quantification is up to four times longer than a pathologists' assessment of hematopoietic cellularity (2 min on a standard laptop computer vs. less than a minute per image for pathologists), it has the advantage of providing quantitative information on several BM compartments at once in a highly reproducible manner.

The quantitative output for the bone, adiposity and interstitium/microvasculature mask in the *MarrowQuant* results is straight-forward. Two results are provided for the quantification of the hematopoietic compartment due to discrepancies in the most appropriate denominator to be used. For users interested in subdividing the BM compartment (e.g., Figures 5–8) the only relevant output for the hematopoietic compartment is the result of Equation 2. For users who would like to use *MarrowQuant* exclusively for quantification of the hematopoietic compartment, similarly to pathologist evaluation of “cellularity,” we propose using Equation 1. Note that datasets with a prominent number of low cellularity samples (<30% cellularity) may benefit from choosing Equation 2 (Figure 7).

The user should be aware of some limitations of MarrowQuant regarding BM segmentation. Firstly, the current version of *MarrowQuant* provides area values which compound the full area of interest. Thus, *MarrowQuant* calculations do not provide a regional gradient of cellularity or adiposity within each bone unless the user manually fragments the bone into separate images. A measure of dispersion across a single bone may be of interest when the marrow compartment is expected to be highly heterogenous. This is the case, for example, in early hematopoietic recovery post-aplasia, where loci of hematopoiesis appear heterogeneously across the marrow space.

Secondly, osteoblasts or bone lining cells with prominent nuclei that have a similar color and texture to the nucleated cells that make up the hematopoietic mask, may be included in the hematopoietic area if detached from the endosteal surface (Figures S5A,E). *MarrowQuant* contains a bone mask dilation feature that will prevent cells in direct endosteal position from being counted in the hematopoietic area. However, if detached, depending on the extent of the artifact and the overall hematopoietic cellularity of the sample, such cells may be misclassified as a significant portion of the hematopoietic mask. In this case, the user should manually exclude them as artifacts. Other non-hematopoietic cells with prominent nuclei, likely including both bone lining cells (45, 46) and stromal cells are expected to be recognized within the hematopoietic mask or fall within the undetected area.

Thirdly, since hematopoietic detection is based on identification of nuclei, the area occupied by adipocytic nuclei may contribute to the hematopoietic compartment (Figures S5B,F). In our dataset, this phenomenon constitutes a systematic bias of <5% (see crossing of *MarrowQuant* values on x-axis, Figure 4D), which is an insignificant contribution to the hematopoietic area in samples of medium to high cellularity, but may add variability in samples of low hematopoietic cellularity and high adiposity.

Fourthly, while the nuclei of megakaryocytes are correctly assigned to the hematopoietic mask, their cytoplasm is often included within the microvascular/interstitial compartment, and sometimes identified as bone area due to their similar texture and color (Figures S5C,G). These mis-assigned bone areas are easily detected as squares within the marrow space, and will be automatically subtracted from the total Ma.Ar. In our dataset, the area of mis-assigned megakaryocytes is minimal, but this may become relevant in other biological contexts where megakaryocytes are significantly enlarged and/or increased in number (e.g., myeloproliferative neoplasms). Finally, the interstitium/microvasculature is well-detected by *MarrowQuant* but may be problematic when the RBCs and/or serous infiltrate are lost upon tissue fixation in specific areas, such as on hemorrhage with leakage of vessels, or in perfused samples. The empty spaces must then be taken as artifacts to avoid them being counted as adipocytes, although the total area of interest is thus reduced to quantify the conserved tissue only. A related inherent bias is the fact that the mid region of the bone (e.g., mid-sagittal sections for the tibiae and mid-longitudinal sections for femurs) often contains the central vein, whose lumen is enlarged due to the fixation-related retraction artifact. In the interest of consistency across the dataset, we have opted to classify the enlarged central lumen as an artifact. Sections across the lumen therefore contain a reduced “total area of interest” for analysis. These are examples of issues that the user should be familiar with, and aware of when interpreting results.

### Adipocyte Fragmentation

Adipocyte quantification on BM sections will be largely dependent on the quality of the sample and absolute values must be interpreted with caution. One caveat is that adipocyte size fragmentation is a comparative measure and *MarrowQuant* may hyper- or hypo-fragment adipocytes where membranes are not clear (Figures S5D,H). In our dataset hypo/hyper-fragmentation does not contribute to the overall adipocytic area as compared to manual classification. However, when membranes are intact, as is more often the case with extramedullary adipose tissue due to the nature of the samples, hypo/hyper-fragmentation is a negligible factor.

In our hands, the adipocyte-only detection function of *AdipoQuant* is somewhat more convenient to apply on extramedullary tissue than other current open-source softwares developed specifically for white adipose tissue. Although the number of adipocytes obtained are similar, the area identified by *AdipoQuant* (or *MarrowQuant* at large) seems truer to size. The fragmentation of the adipocytes lies clearly on the membranes and not within the adipocyte itself when compared to, for example, Adiposoft (Figure S6) (47). Identifying an area of interest before quantification also allows the user to quantify whole tissue sections and to eliminate regions that may be irrelevant for a particular analysis (such as vasculature). Thus, while Adiposoft may give a more accurate total adipocyte count in samples of lower quality, *MarrowQuant* will be truer to the total adipocytic area and distribution. Moreover, the *MarrowQuant* adipocyte fragmentation and separate annotation per adipocyte linked to the flexibility of QuPath, allows the user to easily correct for false positives during the post-analysis validation and eliminate them from the analysis.

### Human Application

We have found that the most important difference affecting *MarrowQuant* results between human and mouse lies in the fact that hematopoietic nuclei are less densely packed in human as compared to mouse marrow. Indeed, in our experience, the cytosolic space of mouse hematopoietic cells is relatively small in histological sections as compared to human samples. Additionally, while mouse marrow samples are encased by cortical bone, human trephine biopsies of a similar size comprise only a small part of the entire bone and thus the marrow space as seen in these histological samples are not fully surrounded by cortical bone. Other processing issues come from visible color differences between mouse and human samples. The potentially duller stain of the human samples has consequences on the efficiency for the thresholding methods used in the code and can sometimes result in mis-segmentation. The observed color difference may be due to the nature of the samples, the preparation and staining protocols, and/or subsequent storage of slides before image acquisition. The method for decalcification of the bone may be different which may influence bone staining. Additionally, human slides were imaged weeks after staining whereas mouse image were usually scanned days after staining. These points could explain the observed color alteration in human samples. Technical adaptations are ongoing for an improved quantification of human BM in a later *MarrowQuant* release. In conclusion, due to the different nature of human biopsies compared to mouse samples, several parameters of the plugin must still be optimized to obtain accurate and reliable measurements, which will be the subject of a separate study and a new software release.

## Conclusion

H&E is the most common and routinely performed stain in histopathology. *MarrowQuant* is a semi-automated digital pathology tool and workflow for QuPath, that quantifies four detectable BM compartments (adipocytic, hematopoietic, osseous, interstitial/microvascular, interstitium/microvasculature) and the remaining undetected area, if any, on H&E stained sections of whole intact murine bones. *MarrowQuant* was optimized for paraffin-stained sections but could potentially be applicable to plastic-embedded samples that are also widely used for histomorphometry. Despite some discordances in mid-cellularity samples which also present a high inter-observer variability, *MarrowQuant* cellularity calculation falls within the range of pathologists' estimations.

While the paraffin sections present only one slice of the whole organ, in our experimental setup with mid-sections of entire bones from C57BL/6 mice, the quantification correlates highly with volumetric quantifications of the contralateral whole bone by μCT combined with osmium-tetroxide staining. We were also able to apply the non-invasive technique of MRI *in vivo* to follow changes in fat signal on murine hematopoietic recovery. The results correspond to *MarrowQuant* histological assessment and correlate with blood recovery curves at peak of aplasia. Moreover, *MarrowQuant* provides a size fragmentation distribution of adipocytes and thus may additionally be applied to H&E sections of extramedullary white (omental or inguinal) adipose tissue. This feature has been extracted to offer the possibility of independent use from *MarrowQuant*, and has been named “AdipoQuant.”

Future iterations of the tool applied to human samples could incorporate machine learning methods to overcome the hurdles of the distinct morphology of human tissue and detection of specific cells (e.g., macrophages and megakaryocytes with large cytoplasms, bone-lining cells). Close collaboration with hematopathologists will help to further develop this tool for human tissues and potential clinical research and/or computer-aided diagnostic applications.

## Methods

### Mice

*In vivo* procedures were carried out in accordance with the Swiss law after approval from the local authorities (Service Vétérinaire de l'Etat de Vaud) and experiments were designed according to the ARRIVE guidelines. Eight-weeks old C57BL/6J were purchased from Charles River Laboratories International and maintained at the Center for Studying Living System (CAV) at the EPFL in microisolator cages. Mice were provided continuously with sterile food and water *ad libitum*, and bedding. Mice were housed at room temperature in 12 h light/dark cycles and fed a standard *ad libitum* chow diet.

### Human Samples

This work was performed under the approval of the local ethical authorities (CER-VD). Images of human bone marrow H&E stained paraffin sections from diagnostic samples of patients undergoing treatment for acute myeloid leukemia were received for analysis as blinded, anonymous images form the Institute of Pathology at Lausanne University Hospital (CHUV). The trephine biopsies were processed for standard pathology diagnosis and H&E stained paraffin sections were scanned using a 40x objective.

### Bone Marrow Transplantation

Eight-weeks-old female C57BL/6 mice were lethally irradiated with a total 850 rad dose in an X-ray radiator (RS-2000, RAD SOURCE) 24 h before transplant. The dose was split in two doses of 425 rads separated by a 4-h interval. Total bone marrow cells were isolated from crushed bone marrow of 8-weeks-old female C57BL/6J donor mice in phosphate buffered saline (PBS, Life Technologies 10010056) solution with 1 mM EDTA (Thermo Fisher Scientific 15575020). Total BM cells for transplantations by SR-S were obtained by flushing instead of crushing. RBCs were removed by incubation with ice-cold lysis buffer (BioLegend) for 30 s. Samples were filtered through a 70 μm cell strainer (Sigma Aldrich CLS431751) and centrifuged at 300 g for 10 min at 4°C. Recipient mice were injected with 125,000 donor cells via tail-vein injection. For at least 2 weeks after lethal irradiation mice were treated with paracetamol and antibiotics in the form of 30 mg of Enrofloxacin (300 μl of Baytril 10% solution, 100 mg/ml, Bayer) and 125 mg of Amoxicillin (2.5 ml of Amoxi-Mepha 200 mg/4 ml, Mepha Pharma AG) as well as 500 mg of Paracetamol (Dafalgan^®^) to 250 ml of drinking water protected from light. Peripheral blood was collected to assess blood recovery (blood volume 50 μl) and analyzed by standard veterinarian blood cell counter (ABC™). Mice were sacrificed by CO_2_ inhalation.

### Magnetic Resonance Imaging

All experiments were conducted on a 9.4T/26 cm horizontal magnet (Agilent/Varian) using a home-built quadrature transceiver of 20 mm diameter loops (Figure S7). Animals were anesthetized with 1.5% of isoflurane, respiration, and temperature were monitored during the whole study. Animals were placed on their side and the leg was immobilized in a contracted position. A triple point Dixon acquisition (39) was performed using a spin-echo based sequence (te = 9 ms, tr = 1.5 s) with 10 coronal slices of 0.8 mm on a FOV 25 × 20 mm^2^ (acq. Matrix 192 × 128). Dixon's technique in combination with a spin-echo based sequence minimized signal loss of the marrow due to bone magnetic susceptibility. Three acquisitions were performed with the echo acquisition shifted 0, 0.4, and 0.8 ms corresponding to the fat-water chemical shift of 1,250 Hz at 9.4 T. Images were reconstructed using a homebuilt Matlab script to extract water and fat maps. Phase unwrapping was performed using a toolbox from Matlab file exchange (48). At the end of the MR acquisitions, animals were sacrificed, tibia and femur were dissected to conduct histology and validate MR Dixon acquisitions.

### Histology

Bones were extracted and cleaned of soft tissue. Long bones and cranium were placed loosely in histology cassettes while spine (including tail) and paws were placed in histology cassettes (Simport M505-11) with sponges (Simport M476-1) to flatten the samples for facilitated sectioning. Bones were fixed for 24 h at room temperature in 10% neutral buffered formalin (VWR 11699404), then rinsed three times with phosphate buffered saline (1x PBS) solution. Bones were decalcified in 20% sodium-citrate (Sigma Aldrich 71405) and formic acid (ROTH 4724.3) solution (v/v) for 30–36 h or in 0.4 M EDTA pH 7.4 (Sigma Aldrich 607-429-00-8) for 2 weeks (solution changed every 3 days) at room temperature. The two different decalcification protocols resulted in similar quality of H&E-stained paraffin sections. Bones were washed three times in PBS or under running tap water for 2 h before transferring to 70% ethanol (Reactolab 96170). The tissues were submitted for stepwise dehydration and embedded in paraffin blocks for sectioning at 3–4 μm thickness with a rotary microtome (RM, Leica microsystems). All cuts were mid-bone sections (i.e., mid-longitudinal for femurs and mid-sagittal for tibiae). Bone silhouettes that do not correspond to those presented in Figures 5, 6 for mid-bone sections were discarded. For mid-longitudinal sections of the femur, at the proximal end, both the head and the neck should be visible, while at the distal end, the two condyli (medial and lateral) should be visible. For mid-sagittal sections of the tibia, at the proximal end, the tibial plate should be intact with conservation of the tuberosity and crest of the tibia; at the distal end, insertion of the fibula as well as the lateral and medial malleolous at the ankle joint should be visible. Femoral and tibial silhouettes that preserve these features should then preserve a contiguous marrow cavity. After floating on a water bath to flatten, sections were mounted on glass slides (Superfrost+ slides, Menzel gläser). Paraffin sections were stained with Hematoxylin and eosine (H&E) using the Tissue-Tek Prisma automate (Sakura) and permanently mounted using the Tissue-Tek glass G2-coverslipper (Sakura) to assess morphology.

### Image Acquisition and Processing

Whole-slide images were acquired with an automated slide scanner (Olympus VS120-SL) at 20x magnification with focus points along the total area of interest or using the *in-situ* focus mode using the accompanying software (Olympus VS-ASW L100 2.9). VSI files obtained from scanning were directly loaded into QuPath for analysis on Windows or Mac operating systems. Extracted TIFF files were also used for analysis. The “training-set” consisted of 79 images of full or partial bones. The “validation-set” consisted of 59 images of full bones. Parameters are pre-set but adjustable by the user if deemed necessary (e.g., adipocyte circularity or size, see tutorial for details). The software workflow follows basic morphological operations, color deconvolutions, thresholding, smoothing operations, and watershed as annotated in the code provided as open source and described in the technical guidelines. When accounting for pre-processing steps such as the drawing of the regions of interests as well the as the segmentation process, it typically takes 1–2 min in order to obtain *MarrowQuant* outputs for an image with a sample of a mouse bone. Technical details are annotated in the code and details on download, installation and use of the plugin with the QuPath software are given in the tutorial (*MarrowQuant* Tutorial, Supplementary Material).

### MicroCT

Fixed bones were scanned for reconstruction of undecalcified bone. Samples were placed in an Eppendorf tube with PBS and scanned using a Quantum X-Ray Micro-CT Scanner (Perkin Elmer Quantum) at 90 kV, 160 μA, CT 160, live 80, and field of view (voxel size) 20 μm^2^ at setting “Fine” for 2 min per sample. A hydroxyapatite phantom was included as control. The software suite provided by the manufacturer was used for image acquisition and reconstruction. Bones were then processed further with formic acid decalcification and osmium tetroxide staining.

#### Osmium Tetroxide Staining

Decalcified bones were rinsed in distilled water. Osmium tetroxide solution was prepared fresh (1% osmium tetroxide [Electron Microscopy Sciences 19110), 2.5% dichromate potassium solution (VWR 1.04864.0500)] with appropriate chemical safety protection and samples stained in 20 ml glass scintillation vials (Electron Microscopy Sciences 7632) in 2 ml of solution at room temperature for 48 h. Bones were washed with distilled water three times and transferred to Eppendorf tubes with distilled water for μCT acquisition as done previously. Quantifications were done on the Analyzer 10.0 software (Analyze Direct, Inc.).

### Statistical Analysis

Values are shown as mean plus or minus the standard deviation. Statistical analysis was performed using GraphPad Prism (version 8.0.0, GraphPad Software) or R Statistical Analysis Software (2013, R Core Team). Mean values were compared by Student's *t*-test or two-way analysis of variance (ANOVA). Statistical significance was accepted for *P* < 0.05, and reported.

## Data Availability Statement

A full package of MarrowQuant and AdipoQuant codes is available on GitHub (https://github.com/Naveiras-Lab/MarrowQuant/tree/qupath-0.1.4), along with a detailed tutorial to download QuPath 0.1.4 and install MarrowQuant and AdipoQuant plugins, as well as the Image Data Resource link and accession number to the full dataset and image bank [IDR, (49)].

## Ethics Statement

The studies involving human participants were reviewed and approved by Comission cantonale d'étique de la recherche sur l'être humain du Canton de Vaud (CER-VD). Written informed consent for participation was not required for this study in accordance with the national legislation and the institutional requirements. The animal study was reviewed and approved by Service de la consommation et des affaires vétérinaires du Canton de Vaud (SCAV).

## Author Contributions

ON and JT conceived ideas, designed experiments, analyzed results, and wrote the manuscript. JT performed all experiments and analyses unless otherwise stated. CB, OB, and RSark conceptualized, designed, and implemented the code. DB, TK, RSark, and OB edited and migrated the code to the final version. DB, RSark, and FS performed histological quantifications. Pathologists BB, RSarr, VN, LL, and CB evaluated histological sections for hematopoietic cellularity, provided valuable feedback, and discussions. NK customized a coil and protocols for imaging, and performed MR imaging and analyses. DT contributed to microCT analysis and blood recovery curves. SR-S, FS, DT, and VC contributed to specific transplants and histological mounting/analysis. ES provided slides and discussions on histology. ON initiated the project. All authors edited and reviewed the final manuscript.

## Conflict of Interest

The authors declare that the research was conducted in the absence of any commercial or financial relationships that could be construed as a potential conflict of interest.
