# Supplementary material for: MarrowQuant Across Aging and Aplasia: A Digital Pathology Workflow for Quantification of Bone Marrow Compartments in Histological Sections
Source: Front Endocrinol (Lausanne). 2020 Sep 24;11:480. doi: 10.3389/fendo.2020.00480 (PMC7542184; doi:10.3389/fendo.2020.00480)
Supplement: Supplementary file 2 [file Data_Sheet_1.pdf]

Supplementary Material

MarrowQuant Tutorial

The following supplementary information supports code for the usage of the designed plugin. A full package of *MarrowQuant* and *AdipoQuant* codes is available on GitHub along with a detailed tutorial to download QuPath 0.1.4 and install MarrowQuant and AdipoQuant plugins as well as the link to the full dataset and image bank: https://github.com/Naveiras-Lab/MarrowQuant/tree/qupath-0.1.4.

MarrowQuant

MarrowQuant is a user-friendly algorithm for the quantification of H&E bone marrow tissue biopsies in whole slide images, implemented as a series of QuPath Scripts.

Keywords: Digital Pathology, Whole slide Imaging, QuPath, Bone Marrow

Installing & Running *MarrowQuant*

MarrowQuant is a script initially developed as a plugin integrated in the image processing software Fiji/ImageJ and adapted afterwards for QuPath using Fiji as an extension and groovy language for scripting. We will first outline QuPath installation steps, and then we will describe how to use *MarrowQuant* within QuPath.

The current version of *MarrowQuant* in QuPath 0.1.4 version has been extensively tested in mouse bone marrow sections; no information exists on accuracy for analysis of other mammalian bones. For human biopsies, preliminary tests show a reasonable accuracy but with systematic errors due to lower hematopoietic cell packing. An adapted version for the analysis of human trephine biopsies is under development with QuPath 0.2.1.

QuPath 0.1.4 Installation

Download and extract the zip file corresponding to your operating system from Zenodo:

https://zenodo.org/record/3949733#.XxaXSZ4zY2x

In the extracted zip archive, you will find within the folder the QuPath executable, as well as an extensions folder that needs to be configured as per the instructions below.

Setting up QuPath 0.1.4 for MarrowQuant

1. Download this repository and navigate to the 'Setup' directory.
2. Add the contents of the 'Extensions' directory in the 'Extensions' directory from the QuPath download.
3. Copy the "ij-plugins" directory to a location and keep track of it.
4. Start QuPath.
5. In Help>Show setup options, make sure to uncheck the verification of updates at launch, as it is not recommended to use more recent versions of QuPath as they may be unsupported by *MarrowQuant*.
6. Set the correct maximum amount of RAM you need. The typical value recommended is around three quarter of the maximum RAM that the computer possesses, depending on your usage of other programs simultaneously with QuPath.

7. Go to Edit>Preferences, and enter the path of the QuPath Extensions folder under "Extensions directory", in "ImageJ plugin directory", enter the path of the "ij-plugins" folder in "Script Directory", enter the path of the "Code" Directory of MarrowQuant

Creation of a project

Users must create a QuPath project in order to work on the images of interest. It is important that you execute the next steps in the same order that is indicated. The image formats compatible with the program are RGB .vsi and .tiff files. Note that a down-sampling of 4 is integrated in the plug-in.

Create an empty folder where you intend to put your project that can be named “Qproject”, for example. In QuPath, in the "Project" tab, click "Create project" and select the empty folder you just created. Now gather all the images you want to work with into a single folder, including other needed folders containing .ets files for example. You can call this folder “Images”. Put this folder in the empty folder where you created your project. Back on QuPath, in the "Project" tab, click "Add images", then click "Choose files". Select the images in the "Images" folder you want to import in the project. Finally, click "Import". Now that your project is created, each image should be displayed in a list in the “Project” tab and should have corresponding "Overview" and "Label" files following it.

As you do not need these files, the easiest way to remove all of them at once is to use a script we provide for that purpose. In Automate>Shared scripts, click on the "Remove Overview And Label" script. This will open the script editor and display the script you selected. Click Run>Run. A few arbitrary error messages may pop up after running the script but can be disregarded. Now your project should only contain the corresponding images without their Overview and Label equivalents.

Pre-processing of an image

Before running the main script of *MarrowQuant* in QuPath, the user must first design the regions of interest needed for the program to work.

We recommend having a look at Dr. Pete Bankhead’s series of Youtube QuPath tutorial videos and on our tutorial video on the following link: https://www.youtube.com/watch?v=_Sepnr9Cg_0. It will prove very useful to become familiar with the QuPath interface and understand how the different tools available in QuPath to design regions of interest work. Several basics tips and guidelines for drawing regions can be found in the *User technical tips* file. In the "Annotations" tab, you can find the different classes that are possible to assign to an object you drew. Those classes are called annotations. The three annotations that are needed in order for *MarrowQuant* to work are the *Tissue Boundaries*, *BG* (which stands for Background), and *Artifact*. Capitalization is important

It could be useful to enable and conversely disable some QuPath features.

First, users can disable the View>"Show slide overview" function. A useful function to enable is View>"Fill annotations", which will make it easier to visualize the shape of your different annotations.

As we think the Wand drawing tool is very useful, we also recommend changing values at the bottom of Edit>Preferences, to 1.0 and 2.0 for Wand smoothing and Wand sensitivity respectively, which will make it easier to use to establish Tissue Boundaries.

The *Tissue Boundaries* class affects the region that needs to be processed. As the aim of *MarrowQuant* is to segment and quantify bone marrow compartments including the cortical bone region if desired, users should try to exclude other regions than those of the Tissue Boundaries.

The Tissue Boundaries must be locked before proceeding to the next step by: right click>annotations>lock.

The *BG* class is an annotation that is needed for background correction. We recommend to simply draw a small rectangle in a region of the same color of the background. That annotation must be contained in the *Tissue Boundaries* annotation. When drawing the *BG* annotation, users must make sure that the region within the annotation is as homogenous as possible. Even a few pixels of tissue residue of a different color can influence the background correction algorithm.

The *Artifact* annotation is an optional compartment which functions to exclude the affected regions from the processing. Like the *BG* annotation, all of the *Artifact* annotations must be strictly contained within the Tissue Boundaries, otherwise they may be not considered in the segmentation process. Typical regions affected as *Artifact* are large fixation artifacts. The reason is that those regions may be assigned as adipocytic compartment by *MarrowQuant* since adipocyte ghosts are the same color as the background. If not originally excluded from the *Tissue Boundaries*, other regions such as muscle or cartilage should be affected as *Artifact* as well.

Processing

Once all the annotations (Tissue boundaries, BG and artifacts) are done.

The user can launch the plugin (Automate > shared scripts **MarrowQuant**). Then click "Run">"Run" or "Run">"Run for project" if you want to process multiple images of the same project at the same time.

At the beginning of the script, the user may modify the minimum and/or the maximum size of the adipocytes to be detected. Recommended parameters are in the script by default as minimum size 120µm2, maximum size 5000μm^2, and minimum circularity 0.3. This is a very permissive setting and users are welcome to restrict these parameters further once *MarrowQuant* has been visually validated in their dataset.

Post-processing

Once all the images needed in a certain project have been processed, you need to export the outputs from QuPath. A specific script was made in order to do that for the current project opened in QuPath.

Simply open the script *MarrowQuant_Export_Results* and click "Run">"Run for project", and select the images from which you want to export the results.

The resulting outputs can be found in the "results" folder, found in the project folder.

The results are exported as a .txt format, but upon opening the file, the user can simply copy everything from the file and paste it in an excel sheet for further analysis.

MarrowQuant user guidelines

Below is a list of items the user should be paying attention to when designing the *Tissue Boundaries* and *Artifacts* regions:

The main reason it is recommended to the user to manually design what we call "artifact" regions, is to reduce the detection of false positive adipocytes by the program.

Adipocyte ghosts are the same color as the background and non-selected artifacts are often fragmented by the watershed algorithm and thus not selected by the maximum adipocyte size filter. Therefore, as many fixation artifacts as possible must be selected as Artifacts.

The segmentation of the bone compartment includes an automatic thresholding step. This step will work optimally when images contain all bone staining homogeneously appearing as expected in standard histology samples.

Given the type of automatic threshold we use, it means that a significant difference in bone color, for example if the bone appears as very bright, might result either in a mis-segmented bone compartment, or in detection of bone in regions where there is none. Conversely, very darkly stained bone can be mis-detected as nucleated cells. The best workaround is to work with as high-quality slides and H&E staining as possible, and to exclude as much of the mis-colorings from the *Tissue Boundaries* region as possible for optimal thresholding.

Muscle will generally be assigned as "interstitium" by the program. Hence our suggestion to the user to either not include muscle in the Tissue Boundary, or to assign it as artifact.

In rare cases, cartilage may be assigned as hematopoietic compartment. When it happens, the user should evaluate whether the incidence on the results is negligible or significant. If significant, it is also very easy for the user to assign cartilage areas as artifacts when required.

QuPath technical tips

Lock / unlock regions

Once you have drawn the Tissue Boundaries region, in order to be able to design artifacts within it, you must lock the Tissue Boundaries region so it cannot be modified. This is done by:

Select the region, by using the Move tool and double clicking the region, or selecting it via the Annotations tab.

Right click > Annotations > lock/unlock

Undo / redo

You can "Undo" actions such as deleting or creating a new region with ctrl+z on the keyboard. To “redo” them back, press ctrl+shift+z.

Note however that you cannot undo a specific modification of an annotation. Performing "Undo" while having a region selected will simply have the effect of removing it

The way the **Wand tool** works is mostly dependent on the sensitivity you set (edit>preferences>Wand smoothing/sensitivity) and the zoom you are at. If you want to be very precise, you may want to zoom closer. If conversely you want to quickly draw big regions, it will be easier when zooming out.

When using either the Brush or the Wand tool, maintaining Alt on the keyboard while clicking will have the effect of erasing instead of drawing. This proves very useful when trying to remove everything that is external to the bone edge with the Wand tool, for example.

AdipoQuant

Installing & Running *AdipoQuant*

The *AdipoQuant* function was devised for the purpose of adipocyte segmentation in extramedullary adipose tissue samples. The script works with QuPath where projects can be created with raw image files (for example .vsi or .tiff formats).

Steps from launch to results:

Create a project in QuPath

1. Define “Tissue Boundaries” and “Artifacts” annotations.
2. Open the script: Automate/Shared scripts/AdipoQuant
3. Press Run to launch the processing.
4. At the beginning of the script, the user may modify adipocyte parameters including the minimum size, maximum size, and minimum circularity of adipocytes.

Recommended values are set by default to minimum size of 300µm2, maximum size of 15,000µm2, and minimum circularity of 0.0. The feature “exclude on edges” is by default set to true.

Export Results

To export results, open and run the AdipoQuant_Export_Results script.

The resulting outputs can be found in the "Results" folder found in the project folder. The results are exported as a .txt format. Upon opening the file, the results can be copied from the file and pasted in an excel sheet to be able to view and sort the data as preferred.
