# Supplementary material for: MarrowQuant Across Aging and Aplasia: A Digital Pathology Workflow for Quantification of Bone Marrow Compartments in Histological Sections
Source: Front Endocrinol (Lausanne). 2020 Sep 24;11:480. doi: 10.3389/fendo.2020.00480 (PMC7542184; doi:10.3389/fendo.2020.00480)
Supplement: Supplementary file 3 [file Data_Sheet_2.pdf]

Supplementary Material

This datasheet includes the text code version of the codes and it is only an illustration and a quick reference for the code logic. To have access to the functional version of the codes, please refer to the following GitHub link:
https://github.com/Naveiras-Lab/MarrowQuant/tree/qupath-0.1.4

MarrowQuant

```
all_regions = getAnnotationObjects()
clearDetections()
def tissues = all_regions.findAll{ it.getPathClass() == getPathClass("Tissue Boundaries") }
tissues.eachWithIndex{ tissue, k ->
def mq = new MQBuilder().assignTissueAnnotation( tissue )
.assignTissueNumber( k )
.assignAdipMin( 120.0 )
.assignDownsample( 4 )
.create()
mq.run()
}
import ij.*
import ij.process.*
import ij.measure.Measurements
import ij.gui.*
import ij.plugin.ImageCalculator
import ij.plugin.Thresholder
import ij.plugin.filter.ThresholdToSelection
import ij.plugin.filter.ImageMath
import ij.plugin.RGBStackConverter
import ij.plugin.RoiEnlarger
import ij.plugin.frame.RoiManager
import sc.fiji.colourDeconvolution.*
import groovy.transform.ToString
import groovy.transform.builder.Builder
import groovy.transform.builder.ExternalStrategy
import qupath.lib.objects.*
import qupath.imagej.objects.*
import qupath.imagej.helpers.*
import qupath.lib.roi.*
import ch.epfl.biop.qupath.utils.*
@ToString(includeNames=true)
class MarrowQuant {
def downsample
def adipMin = 120
def adipMax = 5000
def minCir = 0.3
def excludeOnEdges = false
def testAP = false
def fixNDPIBug = false
def testParams = false
def getIndividualAdipocytes = true
def backgroundAnnotation
def mergedArtifactsAnnotation
def tissueAnnotation
def tissueNumber
```

```
def image
def artifactsRoi
def backgroundRoi
def tissueRoi
def imvRoi
def hematoRoi
def boneRoi
def adipRoi
def ic
def deconvolved
def imvMaskImage
def adipMaskImage
def boneMaskImage
def hematoMaskImage
public void run() {
this.ic = new ImageCalculator()
// Get annotations to use
this.backgroundAnnotation = getAllObjects().find{ it.getPathClass() == getPathClass("BG") }
//tissueAnnotation.getChildObjects()
//tissueAnnotation.getChildObjects().find{ it.getPathClass() == getPathClass("BG") }
this.mergedArtifactsAnnotation = mergeAnnotations(
tissueAnnotation.getChildObjects().findAll{ it.getPathClass() == getPathClass("Artifact") } )
println(this.mergedArtifactsAnnotation)
this.image = GUIUtils.getImagePlus( tissueAnnotation, downsample, false, false )
this.artifactsRoi = getIJRoi( mergedArtifactsAnnotation )
// Do Color Balance
this.backgroundRoi = getIJRoi( backgroundAnnotation )
//this.image = colorBalance( image, backgrounRoi )
this.tissueRoi = getIJRoi( tissueAnnotation )
// Find bone
// Apply Color Deconvolution to get resulting ImagePlus
this.deconvolved = colorDeconvolution( image, "H&E DAB" )
boneIMVFinder()
hematoFinder()
adipFinder()
sendResultsToQuPath()
}
public void boneIMVFinder() {
println( "Bone Finder" )
// Create new image based on stacks 1 and 2
def boneIMVImage = this.ic.run( "Subtract create", this.deconvolved[0], this.deconvolved[1] )
//boneIMVImage.show()
def varianceImage = boneIMVImage.duplicate()
// Create Variance Image
IJ.run( varianceImage, "32-bit", "" )
IJ.run( varianceImage, "Variance...", "radius=1")
IJ.run( varianceImage, "Add...", "value=1")
//IJ.run( varianceImage, "Smooth")
// IB
// BUGFIX 19.01.2016 with Oli
// if needed to fix NDPI bug
if( fixNDPIBug ) {
// extra step where we set the Bone IMV background to Not A Number before division
def boneProc = boneIMVImage.getProcessor()
boneIMVProc.setRoi( this.tissueRoi )
```

```
boneIMVProc.setAutoThreshold("Huang Dark")
Roi sel = ThresholdToSelection.run( boneIMVImage )
def inverse = sel.getInverse( boneIMVImage )
boneIMVProc.setRoi( inverse )
boneIMVProc.resetThreshold()
}
// END BUGFIX
def boneImage = this.ic.run("Divide create 32-bit", boneIMVImage, varianceImage )
def boneProc = boneImage.getProcessor()
boneProc.setRoi( this.tissueRoi )
boneProc.setAutoThreshold("Li dark no-reset")
def boneMaskProc = boneProc.createMask()
this.boneMaskImage = new ImagePlus("Bone Mask", boneMaskProc )
this.boneMaskImage.setCalibration( image.getCalibration() )
IJ.run( this.boneMaskImage, "Options...", "iterations=12 count=2 black pad do=Close");
IJ.run( this.boneMaskImage, "Options...", "iterations=7 count=1 black pad do=Dilate");
boneMaskProc.setBackgroundValue(0)
// Clear the artifacts
boneMaskProc.fill( this.artifactsRoi )
// Clear the tissue
boneMaskProc.fillOutside( this.tissueRoi )
this.boneRoi = getRoiFromMask( this.boneMaskImage )
this.boneMaskImage.setRoi( this.boneRoi )
//boneMaskImage.show()
// Generation of the IMV compartment
def varianceProc = varianceImage.getProcessor()
// Clear outside Tissue
varianceProc.fillOutside( this.tissueRoi )
//Clear the Bone area
varianceProc.fill( this.boneRoi )
varianceProc.setRoi( this.tissueRoi )
varianceProc.setAutoThreshold("MaxEntropy dark no-reset")
def imvMaskProc = varianceProc.createMask()
this.imvMaskImage = new ImagePlus("IMV", imvMaskProc )
this.imvRoi = getRoiFromMask( imvMaskImage )
//this.imvMaskImage.setRoi( this.imvRoi )
//imvMaskImage.show()
}
def hematoFinder() {
def hematoImage = this.ic.run( "Subtract create", this.deconvolved[2], this.deconvolved[0] )
def hematoProc = hematoImage.getProcessor()
hematoProc.smooth()
hematoProc.setRoi( this.tissueRoi )
hematoProc.setAutoThreshold("Huang dark")
def hematoMaskProc = hematoProc.createMask()
this.hematoMaskImage = new ImagePlus("Hemato Mask", hematoMaskProc )
this.hematoMaskImage.setCalibration( image.getCalibration() )
IJ.run( this.hematoMaskImage, "Options...", "iterations=1 count=2 black pad do=Erode") //
best with Li
// Clear outside Tissue
hematoMaskProc.fillOutside( this.tissueRoi )
//Clear the Bone area
hematoMaskProc.fill( this.boneRoi )
hematoMaskProc.fill( this.artifactsRoi )
//Subtract Hemato Mask to IMV Mask (Avoid Overlap)
```

```
this.ic.run("Subtract", this.imvMaskImage, this.hematoMaskImage )
// Redo selection of IMV
this.imvRoi = getRoiFromMask( this.imvMaskImage )
this.hematoRoi = getRoiFromMask( hematoMaskImage )
//this.hematoMaskImage.setRoi( this.hematoRoi )
//this.hematoMaskImage.show()
}
public void adipFinder() {
def hsbImage = this.image.duplicate()
// Convert to HSB
new ImageConverter( hsbImage ).convertToHSB();
def adipImage = this.ic.run( "Subtract create", deconvolved[2], deconvolved[0] );
// Multiply staturation by 8
hsbImage.getStack().getProcessor( 2 ).multiply( 8 ) //Oli: This completely saturates the image
and should be considered bad practice
this.ic.run( "Add", adipImage, new ImagePlus( "Saturation",
hsbImage.getStack().getProcessor( 2 ) ) )
//adipImage.show()
def adipProc = adipImage.getProcessor()
adipProc.setThreshold( 0, 250, ImageProcessor.NO_LUT_UPDATE ) // Oli: Was 127 then 200
in human
def adipMaskProc = adipProc.createMask()
// Makes sure that the particle analyzer will act within the Tissue Boundaries only and
// with the exception of the bone and artifacts
// Clear outside Tissue
adipMaskProc.fillOutside( this.tissueRoi )
//Clear the Bone area
adipMaskProc.fill( this.boneRoi )
adipMaskProc.setBackgroundValue( 255 )
adipMaskProc.fill( this.artifactsRoi )
adipMaskProc.setBackgroundValue( 0 )
// Morphological operations needed to segment individual adipocytes
def adipMaskImage = new ImagePlus( "Adip Mask", adipMaskProc )
adipMaskImage.setCalibration( this.image.getCalibration() )
IJ.run( adipMaskImage, "Watershed", "")
IJ.run( adipMaskImage, "Options...", "iterations=50 count=5 pad do=Dilate" )
adipMaskImage.show()
//IJ.setAutoThreshold( adipMaskImage, "Default" )
//Performs an enhanced version of Fiji's analyze particle
//IJ.log("Values: "+this.adipMin+", "+this.adipMax+", "+this.minCir)
IJ.run( adipMaskImage, "Invert LUT", "");
IJ.run( adipMaskImage, "Extended Particle Analyzer", "area="+this.adipMin+"-
"+this.adipMax+" circularity="+this.minCir+"-1.00 roundness=0.36-1.00 show=Masks redirect=None
keep=None display" )
//hide()
// Create Selection from the mask
def adipMaskImage2 = IJ.getImage()
adipMaskImage2.unlock()
//adipMaskProc.fill( this.artifactsRoi ) Oli Artifacts are already filler here
//adipMaskImage.unlock()
//adipMaskImage.changes = false
//adipMaskImage.close()
//this.adipMaskImage = adipMaskImage2.duplicate()
adipMaskImage.hide()
//IJ.run( adipMaskImage2, "Invert", "")
```

```
IJ.log("Getting ADIP Mask")
this.adipRoi = getRoiFromMask( adipMaskImage2 )
//this.adipRoi = RoiEnlarger.enlarge( this.adipRoi, -1 )
//adipMaskImage2.setRoi( this.adipRoi )
//adipMaskImage2.show()
// STEP 3.1: Same as STEP 2.1, but preventing overlap of IMV with adipocytes
this.ic.run("Subtract", this.imvMaskImage, adipMaskImage2 )
//this.imvMaskImage.show()
// REDO SELECTION
this.imvRoi = getRoiFromMask( this.imvMaskImage )
// STEP 3.2: Same as STEP 3.1, but preventing overlap of Hematopoietic cells with adipocytes
this.ic.run("Subtract", this.hematoMaskImage, adipMaskImage2 )
// this.hematoMaskImage.show()
// REDO SELECTION
this.hematoRoi = getRoiFromMask( this.hematoMaskImage )
this.adipMaskImage = adipMaskImage2.duplicate()
//this.adipMaskImage.show()
}
public sendResultsToQuPath() {
// QuPath Classes and Colors for IMV (interstitium & microvasculature), Adipocytes,
HematoCells and Bone
def imv_class = getPathClass( 'IMV')
def adip_class = getPathClass( 'Adipocytes')
def aadip_class = getPathClass( 'Adipocyte')
def bone_class = getPathClass( 'Bone' )
def hemato_class = getPathClass( 'HematoCells' )
def artifact_class = getPathClass( 'Artifact' )
def bg_class = getPathClass( 'BG' )
def tissue_class = getPathClass( 'Tissue Boundaries' )
// Colors definition
def imv_color = getColorRGB(255, 58, 163)
def adip_color = getColorRGB(255,240,60)
def bone_color = getColorRGB(158, 237, 208)
def hemato_color = getColorRGB(25, 14, 145)
def artifact_color = getColorRGB(0,0,0)
def bg_color = getColorRGB(255,125,50)
def tissue_color = getColorRGB(75,220,145)
// This part handles writing the results to QuPath
// We should have new child objects
IJ.log( "IMV: "+this.imvRoi)
IJ.log( "Hemato: "+this.hematoRoi )
IJ.log( "Bone: "+this.boneRoi )
IJ.log( "Adip "+this.adipRoi )
IJ.log( "Artifact: "+this.artifactsRoi )
def imv = getQuPathPathObject( this.imvRoi, imv_class )
def hemato = getQuPathPathObject( this.hematoRoi, hemato_class )
def bone = getQuPathPathObject( this.boneRoi, bone_class )
def adip = getQuPathPathObject( this.adipRoi, adip_class )
def artifacts = getQuPathPathObject( this.artifactsRoi, artifact_class )
this.tissueAnnotation.addPathObject( imv )
this.tissueAnnotation.addPathObject( hemato )
this.tissueAnnotation.addPathObject( bone )
this.tissueAnnotation.addPathObject( adip )
def area_tissue = this.tissueAnnotation.getROI().getArea()
def area_artifacts = artifacts.getROI().getArea()
```

```
// Get everything calculated by MarrowQuant, all measurements in pixels
// Adipocyte Area
def area_adips = adip.getROI().getArea()
def n_adips = 0 //??
// Hemato Cells Area
def area_hemato = hemato.getROI().getArea()
// Bone Area
def area_bone = bone.getROI().getArea()
// IMV (interstitium & microvasculature) Area
def area_imv = imv.getROI().getArea()
// Area assigned to any of the other compartments
def area_unassigned = area_tissue - area_bone - area_artifacts - area_hemato - area_adips -
area_imv
def denominator1 = area_tissue - area_bone - area_artifacts
def denominator2 = area_adips + area_hemato
// Computing the values need to calculate the outputs, that is the cellularity, adiposity, etc.
def cell1 = 100 * ( area_hemato / denominator1 )
def adipo = 100 * ( area_adips / denominator1 )
def IMVratio = 100 * ( area_imv / denominator1 )
def Unassigned = 100 * ( area_unassigned / denominator1 )
def cell2 = 100 * ( area_hemato / denominator2 )
this.tissueAnnotation.getMeasurementList().clear();
// Add measurements to each Tissue region
def measurements = this.tissueAnnotation.getMeasurementList()
def U = "um"
def px_size = Utils.getPixelSize()
measurements.putMeasurement("Tissue Number", this.tissueNumber)
measurements.putMeasurement("Area Hematopoietic ["+U+"^2]", area_hemato * px_size *
px_size)
measurements.putMeasurement("Area Adipocytes ["+U+"^2]", area_adips * px_size * px_size )
measurements.putMeasurement("Area IMV (interstitium and microvasculature) ["+U+"^2]",
area_imv * px_size * px_size )
measurements.putMeasurement("Area Bone ["+U+"^2]", area_bone * px_size * px_size )
measurements.putMeasurement("Area of Total Tissue ["+U+"^2]", area_tissue * px_size *
px_size )
measurements.putMeasurement("Area Of Artifacts ["+U+"^2]", area_artifacts * px_size *
px_size)
measurements.putMeasurement("Area Unassigned ["+U+"^2]", area_unassigned * px_size *
px_size)
measurements.putMeasurement("% Cellularity (Hematopoietic area/(Marrow area))", cell1)
measurements.putMeasurement("% Adiposity (Adipocytic area/(Marrow area))", adipo)
measurements.putMeasurement("% IMV area (Interstitium & microvasculature/(Marrow area))",
IMVratio)
measurements.putMeasurement("% Unassigned area(Unassigned/(Marrow area))", Unassigned)
measurements.putMeasurement("% Ratio hemato/(hemato+adipo)", cell2)
measurements.putMeasurement("Min Size", adipMin)
measurements.putMeasurement("Max Size", adipMax)
measurements.putMeasurement("Min Circularity", minCir)
// Setting of the colors to all the annotations and detections
imv_class.setColor( imv_color )
adip_class.setColor( adip_color )
bone_class.setColor( bone_color )
hemato_class.setColor( hemato_color )
artifact_class.setColor( artifact_color )
bg_class.setColor( bg_color )
```

```
tissue_class.setColor( tissue_color )
aadip_class.setColor( adip_color )
// Oli update 03.02.2020: Get Adipocytes as individual ROIs / Detections
if ( this.getIndividualAdipocytes == true ) {
def rm = RoiManager.getRoiManager()
rm.reset()
// Run Analyze Particles on the adip mask
//IJ.run( this.adipMaskImage, "Watershed", "")
//this.adipMaskImage.getProcessor().setAutoThreshold("Huang dark")
IJ.run( this.adipMaskImage, "Analyze Particles...", "add")
// Add each ROI as a child of the tissue
def rois = rm.getRoisAsArray() as List
rois.eachWithIndex{ roi, idx ->
this.tissueAnnotation.addPathObject( getQuPathPathObject( roi, aadip_class ) )
}
measurements.putMeasurement( "Total Adipocytes", rois.size())
}
fireHierarchyUpdate()
}
public PathObject getQuPathPathObject( Roi roi, def theClass ) {
def cal = this.image.getCalibration()
def qROI = ROIConverterIJ.convertToPathROI( roi, cal, this.downsample, -1, 0, 0 )
//qROI = ShapeSimplifierAwt.simplifyShape( qROI, this.downsample )
def det = new PathDetectionObject( qROI, theClass )
det.getMeasurementList().putMeasurement("Area "+Utils.um+"^2", roi.getStatistics().area *
cal.pixelWidth * cal.pixelWidth)
return det
}
public Roi getIJRoi( PathObject object ) {
return ROIConverterIJ.convertToIJRoi( object.getROI(), this.image.getCalibration(),
this.downsample )
}
// Use Color Deconvolution Plugin in MarrowQuant
public ImagePlus[] colorDeconvolution ( ImagePlus image, String stain ) {
def cd = new Colour_Deconvolution()
def matList = cd.getStainList()
def mt = matList.get( stain )
def stackList = mt.compute( false, true, image )
// This returns an array of ImageStacks
// Make into an ImagePlus
def imageStack = new ImageStack( stackList[0].getWidth(), stackList[0].getHeight() )
stackList.each { imageStack.addSlice( it.getProcessor( 1 ) ) }
def deconvolved = stackList.collect{ new ImagePlus( image.getTitle()+"-"+stain, it ) }
return deconvolved
}
public Roi getRoiFromMask( ImagePlus image ) {
def proc = image.getProcessor()
proc.setThreshold( 127, 255, ImageProcessor.NO_LUT_UPDATE )
return new ThresholdToSelection().convert( proc )
}
// Reimplemented Color Balance Class
public ImagePlus colorBalance( ImagePlus image, Roi roi ) {
// Make sure it is RGB
if ( image.getType() != ImagePlus.COLOR_RGB ) return null
//Remove ROI before duplication
```

```
image.killRoi()
def image_balanced = image.duplicate()
image_balanced.setTitle( "Color Balanced "+ image.getTitle() )
// Make a 3 slice stack
def ic = new ImageConverter( image_balanced )
ic.convertToRGBStack()
image_balanced.setRoi( roi )
def statOptions = Measurements.MEAN+Measurements.MEDIAN
// Calculate mean/median of each color
image_balanced.setPosition(1) //R
def isR = image_balanced.getStatistics(statOptions)
image_balanced.setPosition(2) //G
def isG = image_balanced.getStatistics(statOptions)
image_balanced.setPosition(3) //B
def isB = image_balanced.getStatistics(statOptions)
def rgb = [isR.mean,isG.mean,isB.mean]
// find largest value.
double maxVal = 0;
int idx = -1;
double scale;
for( def i=0; i<3;i++) {
if (rgb[i] > maxVal) {
idx = i;
maxVal = rgb[i];
scale = 255/maxVal;
}
}
// Remove ROI again to make sure we apply the multiplication to the whole image
image_balanced.killRoi();
rgb.eachWithIndex { val, i ->
image_balanced.setPosition(i+1);
def ip = image_balanced.getProcessor();
//def normVal = maxVal/rgb[i]*scale;
//IJ.log(""+val+", "+rgb[i]+", "+maxVal);
ip.multiply(maxVal/rgb[i]*scale); //Scaling the other channels to the largest one.
}
// Convert it back
def ic2 = new ImageConverter( image_balanced )
ic2.convertRGBStackToRGB()
return image_balanced
}
}
// The lines below allow us to user the Builder Pattern withourh having to declare it in the
MarrowQuant class
@Builder(builderStrategy = ExternalStrategy, forClass = MarrowQuant, prefix = 'assign',
buildMethodName = 'create')
class MQBuilder {
MQBuilder() {
downsample = 4
adipMin = 120
adipMax = 5000
minCir = 0.3
excludeOnEdges = false
testAP = false
fixNDPIBug = false
```

```
testParams = false
getIndividualAdipocytes = true
}
}
```

Export Results

```
import ch.epfl.biop.qupath.utils.*
// Export results from one or many files to a txt file
def tissues = getAnnotationObjects().findAll {it.getPathClass() == getPathClass('Tissue')}
if( tissues.size() == 0) {
tissues = getAnnotationObjects().findAll {it.getPathClass() == getPathClass('Tissue Boundaries')}
}
// Create Output Path
outputPath = buildFilePath( PROJECT_BASE_DIR, 'results' )
// Make directory in case it does not exist
mkdirs( outputPath )
// Give the results file a name
fileName = 'MarrowQuant_Results.txt'
delimiter = '\t'
// Write results in the desired directory to the desired filename
file = new File(outputPath, fileName)
def U = 'um'
def columns = ["Image Name", "Tissue Number",
"Hematopoietic Area ["+U+"^2]",
"Adipocytes Area ["+U+"^2]",
"IMV Area (interstitium and microvasculature) ["+U+"^2]",
"Bone Area ["+U+"^2]",
"Area of Total Tissue ["+U+"^2]",
"Area Of Artifacts ["+U+"^2]",
"Unassigned Area ["+U+"^2]",
"Equation 2(Hematopoietic area/(Marrow area))%",
"% Adiposity (Adipocytic area/(Marrow area))",
"% IMV area (Interstitium & microvasculature/(Marrow area))",
"% Unassigned area(Unassigned/(Marrow area))",
"Equation 1 hemato/(hemato+adipo)%",
"Min Size",
"Max Size",
"Min Circularity",
"Total Adipocytes",
"Num Detections",
"Num Adipocyte"]
Utils.sendResultsToFile( columns, tissues, file )
```

Remove Overview and Labels

```
/*
* Duplicate an existing project, putting the new files into a subdirectory of the current project.
*
* All image analysis is reset, *except* for any TMA cores. These are kept, along with any metadata.
*
* This is particularly useful for generating a new TMA project with the same dearrayed cores &
metadata,
* but otherwise everything kept the same.
*/
```

```
import qupath.lib.gui.QuPathGUI
import qupath.lib.gui.panels.ProjectBrowser
import qupath.lib.images.ImageData
import qupath.lib.images.servers.ImageServerProvider
import qupath.lib.io.PathIO
import qupath.lib.projects.Project
import qupath.lib.projects.ProjectIO
import java.awt.image.BufferedImage
// Get the running QuPath instance
def qupath = QuPathGUI.getInstance()
// Get the current project
def project = qupath.getProject()
if (project == null) {
println("No project open!")
return
}
// Removes all the 'overview' images
for (def entry in project.getImageList()) {
if ((entry.getImageName() =~ 'overview') || (entry.getImageName() =~ 'label')) {
project.removeImage(entry)
}
}
sleep(1000)
getQuPath().refreshProject()
ProjectIO.writeProject(project)
fireHierarchyUpdate()
// Write the new project itself
//ProjectIO.writeProject(projectNew)
print("Done! Project written to ")
```

AdipoQuant

```
guiscript=true
// Default inputs
def aadipMin = 300
def aadipMax = 15000
def aminCir = 0.0
def aexcludeOnEdges = true
def adownsample = 2
// Import BIOP library
import ch.epfl.biop.qupath.utils.*
import ij.IJ
import sc.fiji.colourDeconvolution.*
import ij.WindowManager
import ij.plugin.ImageCalculator
import ij.ImagePlus
import ij.process.ImageProcessor
import ij.plugin.frame.RoiManager
import qupath.lib.objects.PathDetectionObject
import qupath.imagej.objects.ROIConverterIJ
import ij.macro.Interpreter
import ch.epfl.biop.qupath.utils.Utils
import ij.gui.Roi
import qupath.imagej.gui.IJExtension
```

```
import qupath.lib.gui.helpers.DisplayHelpers
import qupath.imagej.helpers.*
import qupath.lib.roi.PathObjectToolsAwt
import qupath.lib.objects.PathAnnotationObject
clearDetections()
def tb = getPathClass("Tissue Boundaries", getColorRGB(0,255,255))
def ar = getPathClass("Artifact", getColorRGB(0,255,255))
def adip_class = getPathClass("Adipocyte", getColorRGB(0,255,255))
def mq_classes = [tb, ar, adip_class]
def all_classes = getQuPath().getAvailablePathClasses().toList()
mq_classes.each { the_class ->
if (all_classes.find{ it == the_class } != null ) {
println("Class "+the_class+" already exists")
} else {
all_classes.add(the_class)
println("Creating class "+the_class)
}
}
getQuPath().getAvailablePathClasses().setAll(all_classes)
fireHierarchyUpdate()
def ij = IJExtension.getImageJInstance()
ij.show()
def ad = new AdipocyteDetector()
ad.with {
downsample = adownsample
adipMin = aadipMin*downsample
adipMax = aadipMax*downsample
minCir = aminCir
excludeOnEdges = aexcludeOnEdges
}
def tissues = getAnnotationObjects().findAll{ it.getPathClass().equals( getPathClass("Tissue
Boundaries") ) }
tissues.eachWithIndex{ tissue, i ->
tissue.setLocked(true)
println(sprintf("Analysing Tissue Boundaries #%d", i) )
ad.run(tissue)
}
fireHierarchyUpdate()
def imageName = getCurrentImageData().getServer().getShortServerName()
println("Processing complete for " + imageName );
//ij.quit()
// Main class for Adipocyte Detection
class AdipocyteDetector {
def adipMin = 1000
def adipMax = 50000
def minCir = 0.0
def downsample = 2
public void run(def tissue) {
Interpreter.batchMode = true
IJ.run("Close All")
// Returns the image as an ImageJ ImagePlus object with ROIs and the overlay
def image = GUIUtils.getImagePlus(tissue, this.downsample, true, true)
// Pick up pixel size
def px_size = image.getCalibration().pixelWidth
def tissue_roi = image.getRoi()
```

```
image.show()
// Extract ROIs
def overlay = image.getOverlay()
def rois = overlay == null ? null : overlay.toArray() as List
def artifacts = rois.findAll{ it.getName() =~ /Artifact/ } // will always send a List, could be empty
but never null
IJ.log(""+artifacts)
// Merge all artifacts together.
def all_edges = uglyArtifactMerge(artifacts, tissue_roi, image)
// Creates HSB stacks from the original image
def hsb_image = image.duplicate()
IJ.run(hsb_image, "HSB Stack", "")
hsb_image.show()
// Call Color Deconvolution and recover the images (must be done through GUI for now)
IJ.run(image, "Colour Deconvolution", "vectors=H&E hide")
//Pickup the images for later processing
def col1 = WindowManager.getImage( image.getTitle()+"-(Colour_1)" )
def col2 = WindowManager.getImage( image.getTitle()+"-(Colour_2)" )
def col3 = WindowManager.getImage( image.getTitle()+"-(Colour_3)" )
// Creates the final image we are going to process from the hue and brightness image obtained
def ic = new ImageCalculator()
def adip_raw_image = ic.run("Subtract create", col3, col1)
adip_raw_image.show()
image.close()
col1.close()
col2.close()
col3.close()
def saturation = hsb_image.getStack().getProcessor(2) // Saturation is the second image
saturation.multiply(8)
ic.run("Add", adip_raw_image, new ImagePlus("TesT", saturation) )
adip_raw_image.show()
IJ.setRawThreshold(adip_raw_image, 0, 127, null);
// Morphological operations and analyze particle
def adip_mask = new ImagePlus("Adip Mask", adip_raw_image.getProcessor().createMask()) //
IB?
IJ.log( ""+adip_mask.isInvertedLut() )
if( adip_mask.isInvertedLut() ) adip_mask.getProcessor().invertLut()
IJ.run( adip_mask, "Invert", "") // IB?
IJ.run( adip_mask, "Watershed", "" )
adip_mask.show()
IJ.run( adip_mask, "Options...", "iterations=50 count=5 pad do=Erode" )
adip_mask.setRoi( all_edges )
//IJ.run(adip_mask, "Make Inverse", "")
IJ.setRawThreshold(adip_mask, 0, 127, null)
IJ.run(adip_mask, "Analyze Particles...", "size="+this.adipMin+"-"+this.adipMax+"
circularity="+this.minCir+"-1.00 show=Nothing exclude add display")
// Merge all adips as a single selection
def rm = RoiManager.getInstance() ?: new RoiManager()
// Save as Detections in QuPath
def adips = rm.getRoisAsArray() as List
rm.runCommand("Reset")
rm.close()
def total_area = 0
// Measurement of adipocytes areas and displaying of the data in QuPath
adips.eachWithIndex{ adip, idx ->
```

```
def qu_adip = ROIConverterIJ.convertToPathROI( adip, image.getCalibration(),
this.downsample, 0,0,0)
def det = new PathDetectionObject(qu_adip, getPathClass("Adipocyte"))
def area = adip.getStatistics().area
det.getMeasurementList().putMeasurement( "Adipocyte Index", idx+1 )
det.getMeasurementList().putMeasurement( "Area "+Utils.um+"^2", area * px_size * px_size
)
tissue.addPathObject(det)
total_area += area
}
tissue.getMeasurementList().clear();
tissue.getMeasurementList().putMeasurement( "Total Adipocyte Area "+Utils.um+"^2",
total_area * px_size * px_size )
Interpreter.batchMode = false
}
// Excludes the artifacts from the ROI we want to process
private Roi uglyArtifactMerge(def artifacts, def tissue_roi, def image) {
if ( artifacts.isEmpty() ) {
return tissue_roi
}
if (artifacts.size > 0) {
def rm = RoiManager.getInstance() ?: new RoiManager()
rm.runCommand("Reset")
artifacts.each{ rm.addRoi(it) }
def all_artifacts
if ( artifacts.size == 1 ) {
all_artifacts = artifacts[0]
} else {
rm.setSelectedIndexes((0..rm.getCount()-1) as int[])
rm.runCommand(image, "OR")
all_artifacts = image.getRoi()
}
rm.runCommand("Reset")
// AND then XOR with tissue
IJ.log(""+all_artifacts)
rm.addRoi(all_artifacts)
rm.addRoi(tissue_roi)
rm.setSelectedIndexes([0,1] as int[])
rm.runCommand(image, "AND")
def overlap_artifacts = image.getRoi()
rm.addRoi( overlap_artifacts )
rm.setSelectedIndexes([1,2] as int[])
rm.runCommand(image, "XOR")
rm.close()
return image.getRoi()
}
}
}
```

Export *AdipoQuant* Results

```
import ch.epfl.biop.qupath.utils.*
selectDetections();
runPlugin('qupath.lib.plugins.objects.ShapeFeaturesPlugin', '{"area": false, "perimeter": true,
```

```
"circularity": true, "useMicrons": true}');
def um = Utils.um
def columns = ["Adipocyte Index", "Parent", "Area "+um+"^2"]
def resultsfolder = buildFilePath(PROJECT_BASE_DIR, "results")
mkdirs( resultsfolder )
def resultsfile = new File(resultsfolder, "adipocyte-measurements.txt")
println(resultsfile.getAbsolutePath())
def detections = getDetectionObjects()
Utils.sendResultsToFile(columns, detections, resultsfile)
println("Completed")
```
