# Supplementary material for: MarrowQuant Across Aging and Aplasia: A Digital Pathology Workflow for Quantification of Bone Marrow Compartments in Histological Sections
Source: Front Endocrinol (Lausanne). 2020 Sep 24;11:480. doi: 10.3389/fendo.2020.00480 (PMC7542184; doi:10.3389/fendo.2020.00480)
Supplement: Supplementary file 4 [file Presentation_1.pdf]

 doi: 10.3389/fendo.2020.00480

Combined Supplementary Figure Legends

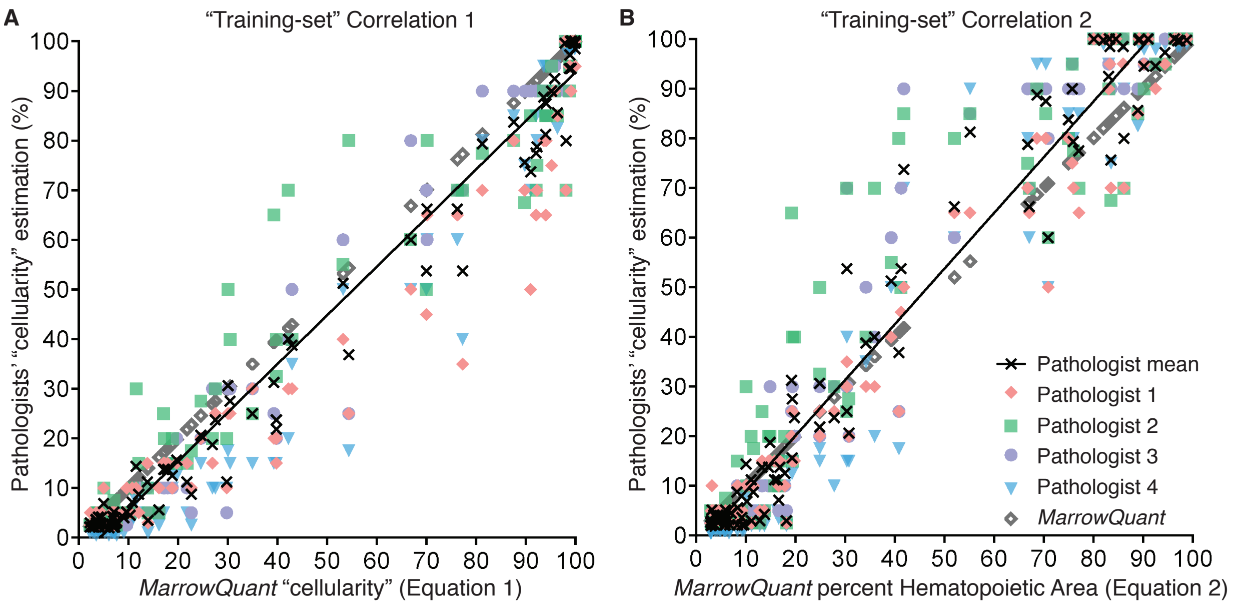

Figure S1. *MarrowQuant* versus pathologists' correlations on the "training-set" of images. **(A)** *MarrowQuant* "cellularity" values (Equation 1) versus pathologists' estimation of "training-set" (pathologists n=4, images n=89, R^2=0.98). **(B)** *MarrowQuant* percent Hematopoietic Area (Equation 2) versus pathologists' "cellularity" estimation of "training-set" (pathologists n=4, images n=89, R^2=0.96). Evaluations done for femurs, tibiae, tail and spine vetebrae of two-month-old female mice at 0 to 25 days post lethal irradiation and total bone marrow transplant. All mice were C57BL/6 housed at room temperature and fed a standard ad libitum chow diet.

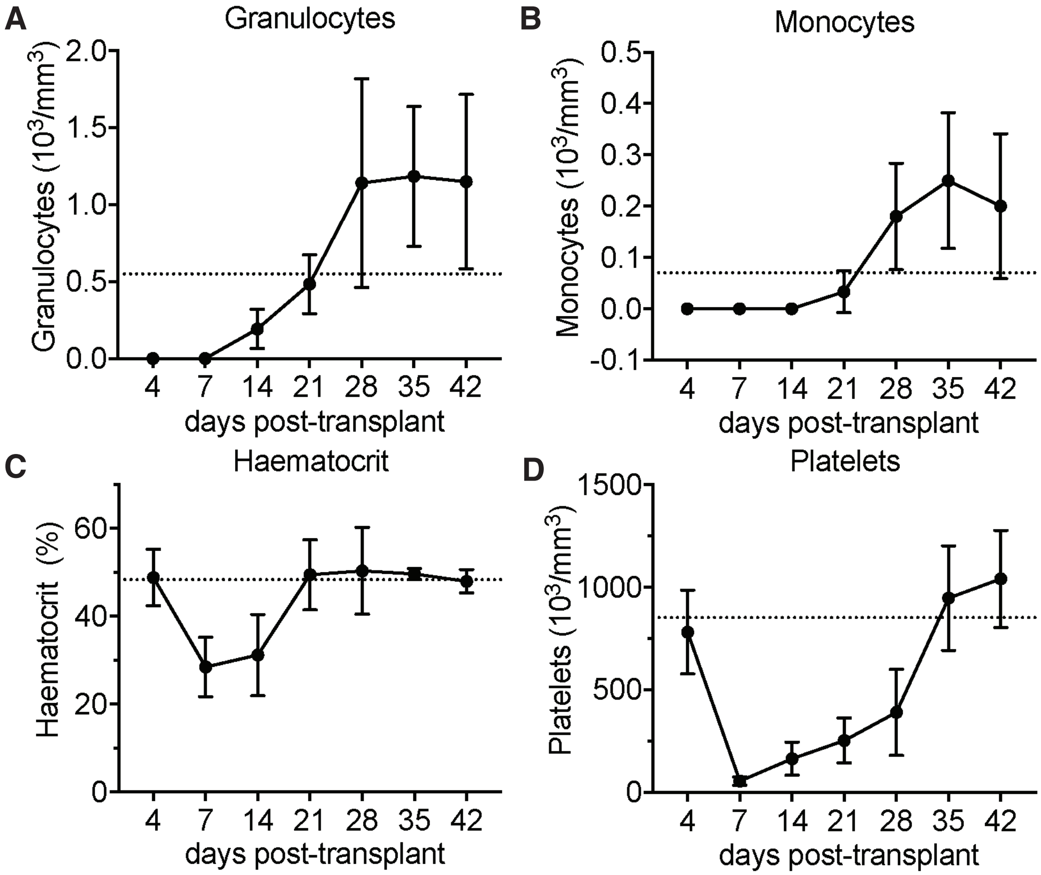

Figure S2. Peripheral blood recovery curves of 8-week-old C57BL/6 female mice having undergone lethal whole-body irradiation and transplantation of 125,000 total bone marrow cells housed at room temperature and fed ad libitum standard chow diet. Recovery of **(A)** granulocytes, **(B)** monocytes, **(C)** hematocrit, **(D)** platelets for the mice presented in Figure 8D (MRI measurements, n=2-13 mice per timepoint). Error bars represent s.d.

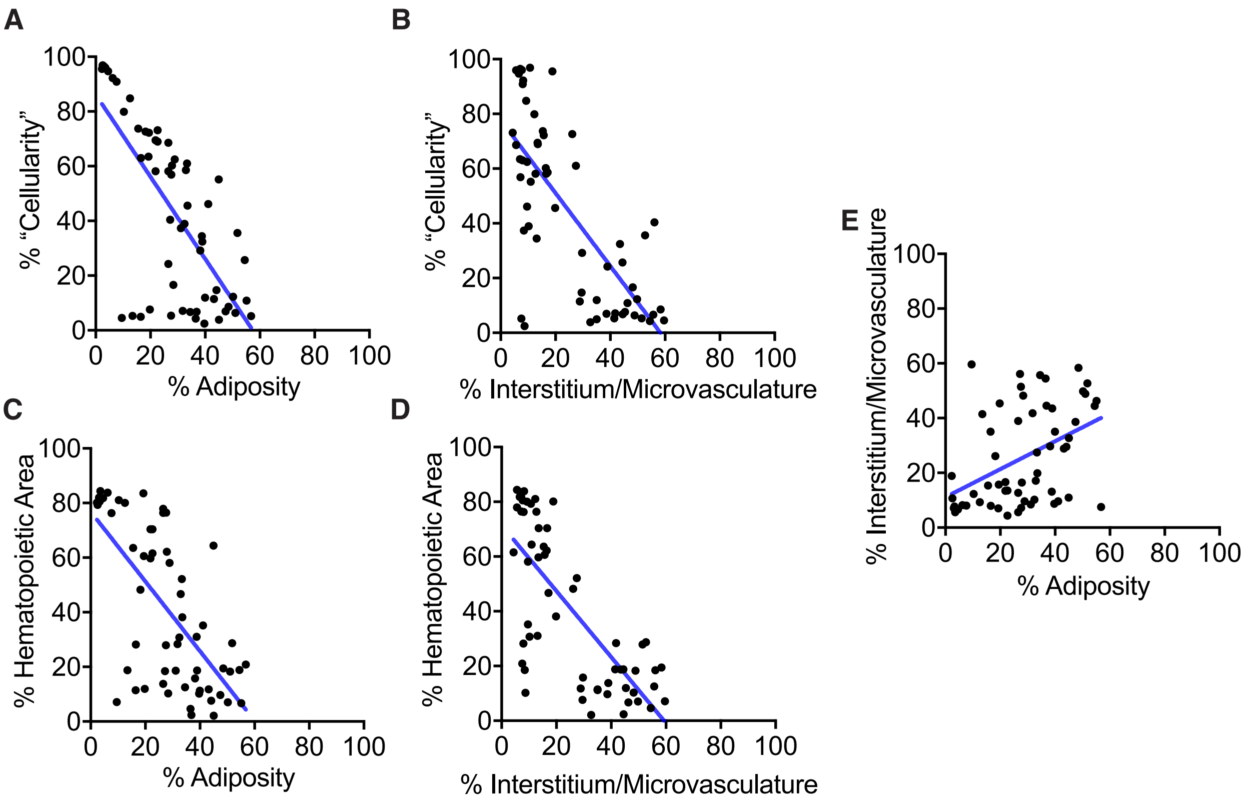

Figure S3 | Reciprocity of the bone marrow compartments as a function of each other, in tibia and femurs (dataset as for Figure 4C and 4D). Note that the non-compressible nature of the bone leads to an interdependency of the three marrow compartments. **a** Percent BM “cellularity” versus BM adiposity (R^2=0.50), **b** percent BM “cellularity” versus percent BM interstitium and microvasculature (R^2=0.54), **c** percent BM hematopoietic area versus percent BM adiposity (R^2=0.45), **d** percent BM hematopoietic area versus percent BM insterstitum and microvasculature (R^2=0.55), **e** percent BM interstitium and microvasculature versus percent BM adiposity (R^2=0.19).

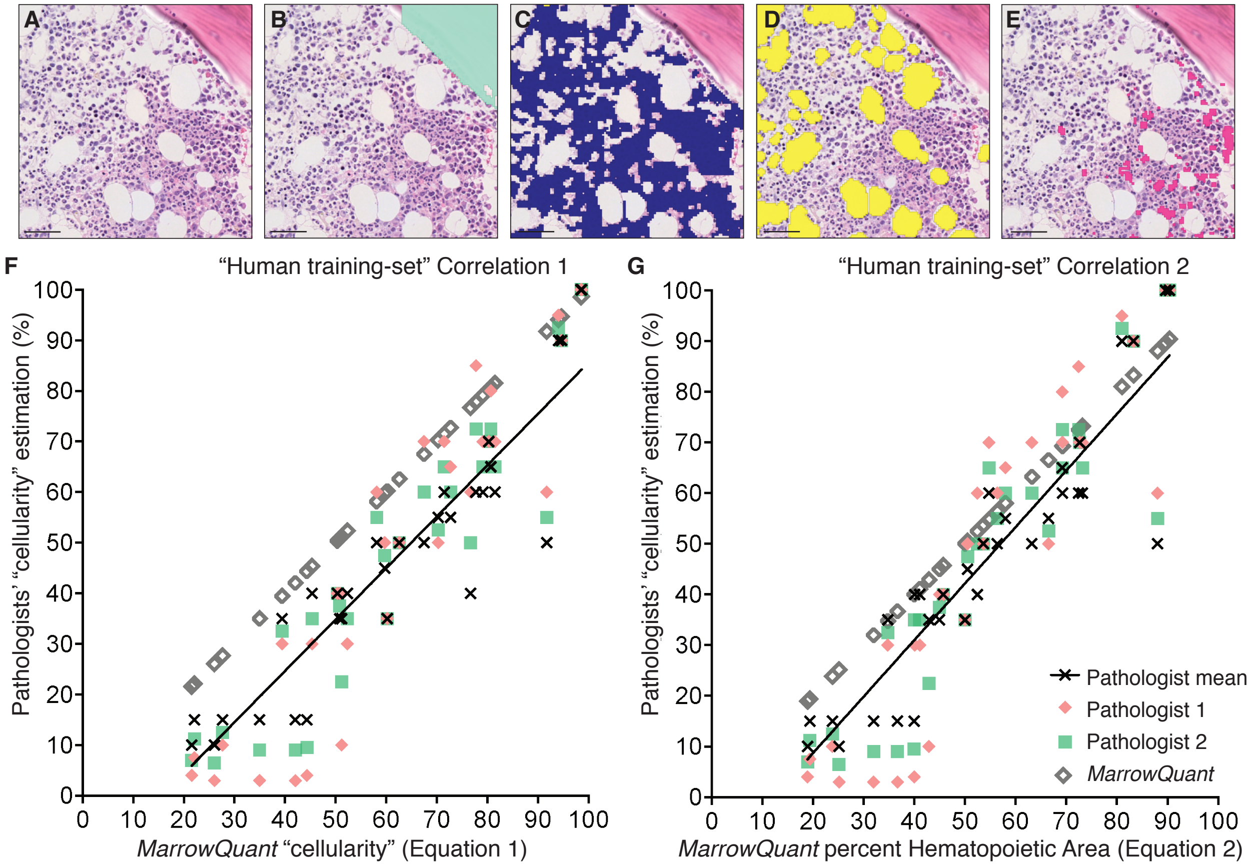

Figure S4. The described version of *MarrowQuant* detects all four BM compartments (**A-E**) in human trephine biopsies, which complements pathologists' evaluation. Diagnosis of example: Acute myeloid leukemia. Scale bars are 50µm. **(F, G)** *MarrowQuant* versus pathologists' correlations on a "human training-set" of images from trephine biopsies of leukemic patients. **(F)** *MarrowQuant* "cellularity" values (Equation 1) versus pathologists' estimation of the "human training-set" (pathologists n=2, images n=32, R^2=0.85). **(B)** *MarrowQuant* percent Hematopoietic Area (Equation 2) versus pathologists' "cellularity" estimation of the "human training-set" (pathologists n=42, images n=32, R^2=0.84). Human-specific variation will be adjusted for in a future *MarrowQuant* release for increased accuracy.

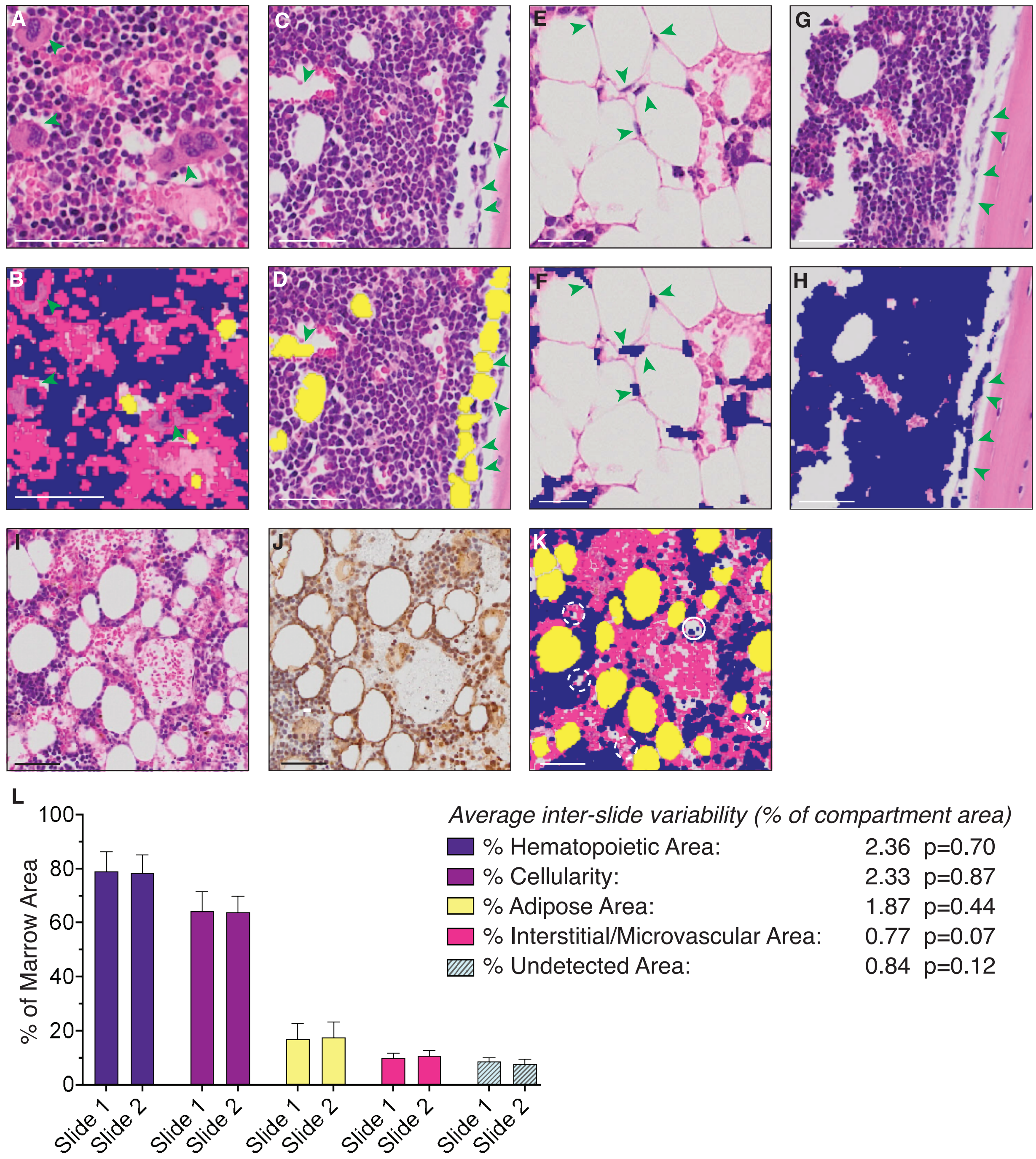

Figure S5. Limitations: Detection errors of *MarrowQuant* attributed to false positive and negative counts. **(A, B, C, D, I)** H&E stained paraffin sections of murine BM. **(E, F, G, H, K)** *MarrowQuant* detection masks for the same images. **(A, E)** Bone-lining cells (arrows) detected as hematopoietic area. Scale bars are 50µm. **(B, F)** Nuclei (arrows) detected as hematopoietic area (purple). Scale bars are 25µm. **(C, G)** The nuclei of Megakaryocytes are detected within the hematopoietic area

(purple), but their cytoplasm (arrows) is often detected as interstitum/microvasculature (pink). Scale bars are 50µm. **(D, H)** Fragmented artifact region (arrows) detected as adipocyte ghosts (yellow). Scale bars are 50µm. **(J)** Perilipin stain for comparison to *MarrowQuant* adipocyte detection. One false-negative adipocyte ghost in **(K)**, not detected by *MarrowQuant,* is indicated with a solid white circle. Note as dashed white circles, adipocyte ghosts that are perilipin positive in **(J)** but morphologically could not have been identified as adipocyte ghosts by *MarrowQuant* or by pathologists as barely visible in the contiguous slide in **(I)**. Scale bars are 50µm. **(L)** *MarrowQuant* error rate between non-consecutive sections of five femurs is insignificant for all compartments by student's paired t-test. Images extracted from samples of Figures 8A and 8B.

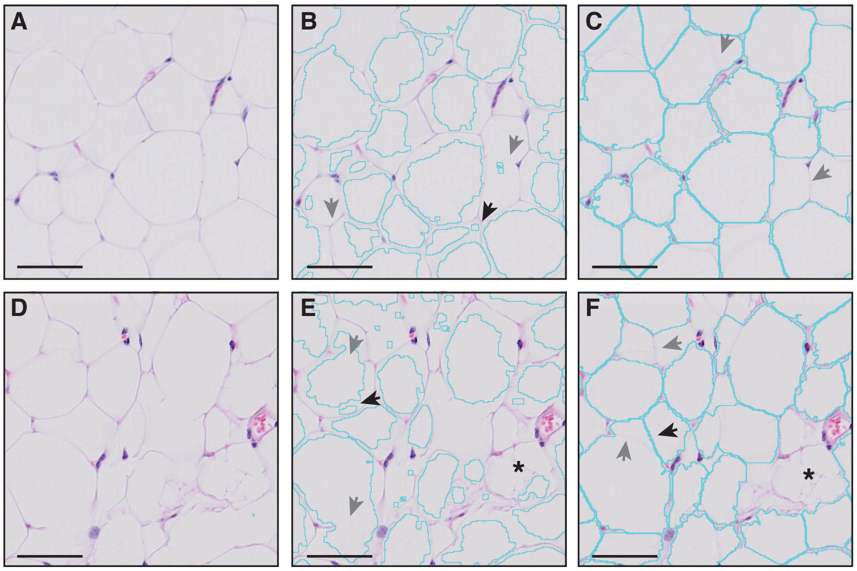

Figure S6. Adipocyte detection as performed by *AdipoQuant* versus available software Adiposoft. **(A, D)** Unprocessed image of adipocyte ghosts, **(B, E)** adipocyte ghost detection by Adiposoft, **(C, F)** *AdipoQuant* detection of adipocyte ghosts. Both scripts detect the majority of adipocytes. None of them detects multilocular adipocytes (e.g. brown adipocytes or immature adipocytes), composed of multiple lipid droplets (*) and may not detect membranes (grey arrows) or hypersegment (black arrows). All images are from H&E stained paraffin sections of omental adipose tissue from two separate (top and bottom panel) ten-week-old C57BL/6 female mice fed a standard ad libitum chow diet housed at room temperature. Scale bars are 50µm. Images extracted from samples of Figure 2C.

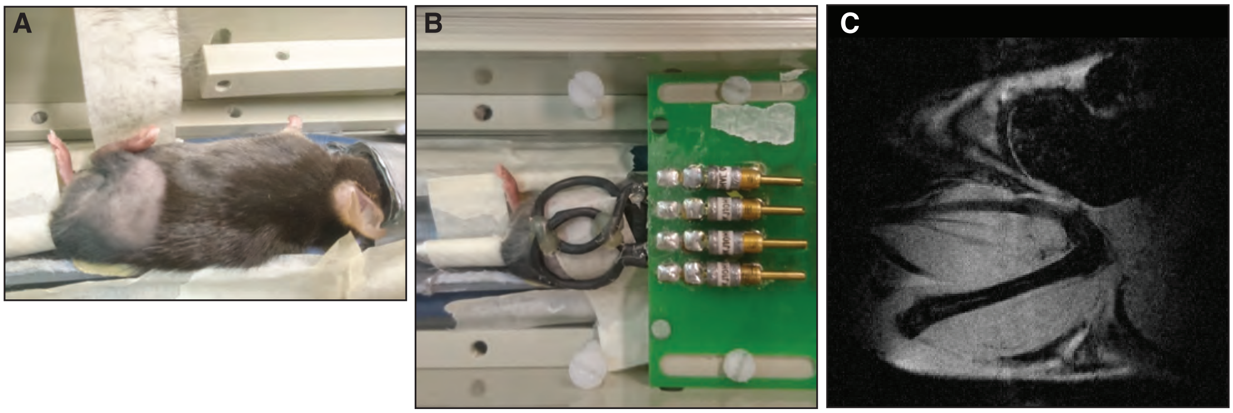

Figure S7. *In vivo* magnetic resonance imaging (MRI) of murine bone marrow. **(A)** The mouse hindlimb is maintained in a fixed position for imaging. **(B)** A home-made magnetic coil is placed over the place of the left femur before MRI acquisition. **(C)** Sample image of a femur with the marrow cavity in view of a C57BL/6 female mouse day 21 post irradiation and bone marrow transplant, housed at room temperature fed a standard ad libitum chow diet.
